# Mechanotransduction pathways in articular chondrocytes and the emerging role of estrogen receptor-α

**DOI:** 10.1038/s41413-023-00248-x

**Published:** 2023-03-03

**Authors:** Ning Wang, Yangfan Lu, Benjamin B. Rothrauff, Aojie Zheng, Alexander Lamb, Youzhen Yan, Katelyn E. Lipa, Guanghua Lei, Hang Lin

**Affiliations:** 1 Department of Orthopaedic Surgery, Xiangya Hospital, Central South University, Changsha, 410008 Hunan China; 2 Department of Orthopaedic Surgery, University of Pittsburgh School of Medicine, Pittsburgh, PA 15219 USA; 3 Department of Bioengineering, University of Pittsburgh Swanson School of Engineering, Pittsburgh, PA 15219 USA; 4 McGowan Institute for Regenerative Medicine, University of Pittsburgh School of Medicine, Pittsburgh, PA 15219 USA

**Keywords:** Physiology, Metabolism

## Abstract

In the synovial joint, mechanical force creates an important signal that influences chondrocyte behavior. The conversion of mechanical signals into biochemical cues relies on different elements in mechanotransduction pathways and culminates in changes in chondrocyte phenotype and extracellular matrix composition/structure. Recently, several mechanosensors, the first responders to mechanical force, have been discovered. However, we still have limited knowledge about the downstream molecules that enact alterations in the gene expression profile during mechanotransduction signaling. Recently, estrogen receptor α (ERα) has been shown to modulate the chondrocyte response to mechanical loading through a ligand-independent mechanism, in line with previous research showing that ERα exerts important mechanotransduction effects on other cell types, such as osteoblasts. In consideration of these recent discoveries, the goal of this review is to position ERα into the mechanotransduction pathways known to date. Specifically, we first summarize our most recent understanding of the mechanotransduction pathways in chondrocytes on the basis of three categories of actors, namely mechanosensors, mechanotransducers, and mechanoimpactors. Then, the specific roles played by ERα in mediating the chondrocyte response to mechanical loading are discussed, and the potential interactions of ERα with other molecules in mechanotransduction pathways are explored. Finally, we propose several future research directions that may advance our understanding of the roles played by ERα in mediating biomechanical cues under physiological and pathological conditions.

## Introduction

Osteoarthritis (OA) is a degenerative joint disease mainly characterized by low-grade inflammation, cartilage degradation, and subchondral bone remodeling, which together lead to pain and disability.^1^ Due to population aging, the incidence of symptomatic OA has increased markedly over the past few decades. According to a Global Burden of Disease study from 2017, among 354 other diseases and injuries, OA is the fifth leading cause of disability worldwide and is therefore the cause of large social health and economic burdens.^2^ Total knee arthroplasty (TKA) is a very successful surgical treatment to reduce knee pain and improve mechanical function for patients with end-stage OA, but it can also be accompanied by several complications, including aseptic loosening, prosthetic joint infection, and periprosthetic fracture. No treatments have been established to slow or reverse OA progression, often resulting in years of increasing pain and disability until TKA is indicated.

There are several risk factors involved in the pathogenesis of OA, such as aging, genetics, obesity, and injury, but biomechanical overload is one of the primary risk factors for OA.^3,4^ Previous research generally supports the conclusion that exercise of moderate intensity and/or most sporting activities that do not cause traumatic injury do not substantially increase the risk of OA.^5,6^ Additionally, clinical trials and in vivo animal studies have shown that proper exercise interventions truly relieve OA pain and improve joint function;^7,8^ therefore, structured land-based exercises are currently recommended as core treatments for knee OA.^9^ Notably, the effects of mechanical force on articular cartilage and chondrocytes depend on the force type, magnitude, frequency, duration, and orientation.

Mechanistically, physical forces are converted into biochemical or electrical signals to induce a cellular response. This special cellular process is called mechanotransduction. New discoveries of putative mechanotransduction elements are quickly expanding our understanding of this unique cell signaling process.^10^ For instance, estrogen receptor α (ERα) has recently been found to play a critical role in governing chondrocyte responses to compressive loading.^11^ Previously, ERα had been shown to be an important mechanical force -mediator in bone formation via the action of a ligand-independent pathway.^12^ Therefore, the novel function of ERα in converting forces into biochemical changes in chondrocytes needs be further investigated. In this review, we first briefly summarize the current understanding of mechanotransduction in chondrocytes and then explore the potential interaction of ERα with other molecules in regulating the chondrocyte response to loading. Finally, we propose several future research directions that can further our understanding of the role of ERα in mediating the cellular response to biomechanical cues under specific physiological and pathological conditions.

## Three-tiered cascade of mechanotransduction pathways in chondrocytes

**Fig. 1:**
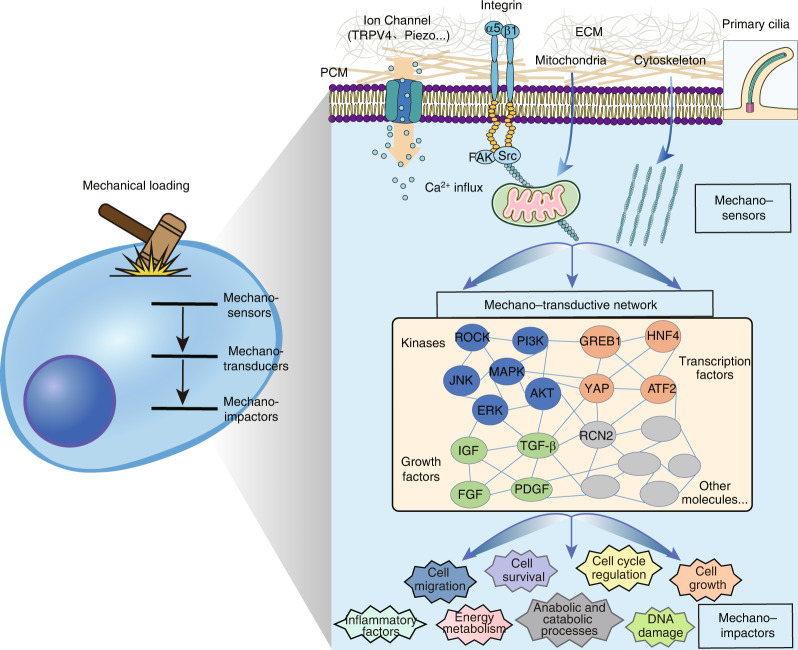
The three-tiered cascade of mechanotransduction pathways in cells. The first structures influenced by mechanical stimulation are different types of mechanosensors, including pericellular matrix (PCM) and extracellular matrix (ECM) components, mechanically gated ion channels and porins (such as TRPV4 and Piezo), integrins, mitochondria, cytoskeleton, and primary cilia. Mechanotransducers include multiple types of factors, such as kinases (ROCK, PI3K-AKT, MAPK, etc.), growth factors (IGF, TGF-β, etc.), transcription factors (YAP, GREB1, etc.), secretory proteins (RCN2, etc.), and other molecules, which are activated by mechanosensors and then alter specific downstream molecules. Mechanoimpactors are the final outputs of mechanical stimulation and cause changes in cell phenotypes and behaviors, such as cell migration, survival, growth, energy metabolism, and inflammation

Previous studies have led to the identification of many cellular structures and molecules that mediate chondrocyte responses to force, including primary cilia, mitochondria, ion channel proteins, transcription factors, inflammatory cytokines, microRNAs, etc.^13,14^ Organizing these molecules based on their major functions would be helpful. To this end, some studies have provided a rough classification of mechanosensitive structures and molecules based on their roles in the mechanoresponses. For example, the nuclear localization of Yes-associated protein (YAP) is influenced by the ion channel protein Piezo1 in neural stem/progenitor cells and osteoblasts, and activated YAP promotes the expression of downstream targets, such as collagen-II.^15,16^ Moreover, recent discoveries are converging on a model in which multiple types of mechanical inputs in a variety of cellular settings influence the conformation and tension of the F-actin cytoskeleton, and then, a change in the cytoskeleton activates transcription factors, such as YAP, to generate downstream biological effects.^17^ Inspired by these studies, we propose three categories based on the principal role of the elements involved in mechanotransduction signaling, including sensors, transducers, and impactors (Fig. 1). Specifically, mechanosensors are defined as the elements that directly respond to mechanical loading by converting mechanical signals into biochemical signals and are primarily composed of cellular structures, including ion channels, cytoskeleton, primary cilia, etc. Mechanoimpactors are the final outputs of mechanical stimulation and can lead to changes cell phenotypes/functions and the extracellular matrix (ECM) compositions and structures; mechanoimpactors include proinflammatory cytokines, energy metabolism factors, and cell cycle regulators.^18–20^ Due to the widespread range of mechanoimpactor effects on cellular activity, mechanoimpactors are not discussed separately herein. In contrast, they are described in the context of specific sensors or transducers. Mechanotransducers are molecules for which clear upstream signaling leads to their activation and can also alter specific downstream molecules. Mechanotransducers connect the actions of mechanosensors and mechanoimpactors; they include kinases and transcription factors. A representative example demonstrating a three-tiered cascade is the Piezo protein–YAP–collagen axis. Piezo proteins (Piezo1 and Piezo2) are nonselective Ca^2+^-permeable cation channels that are also mechanosensors in various cell types.^21,22^ As a transcription factor, YAP has been shown to be a major downstream mechanotransducer activated by Piezos in osteoblasts, in which it regulates the expression of type II and type IX collagen in osteoblasts.^15^ Despite the conceptual simplicity of the three-tiered cascade in chondrocyte mechanotransduction, certain elements may exhibit more than one function. For example, mitochondria can be sensors, mediators, or impactors in mechanotransduction pathways.

### Mechanosensors of chondrocytes and their roles in OA pathogenesis

**Fig. 2:**
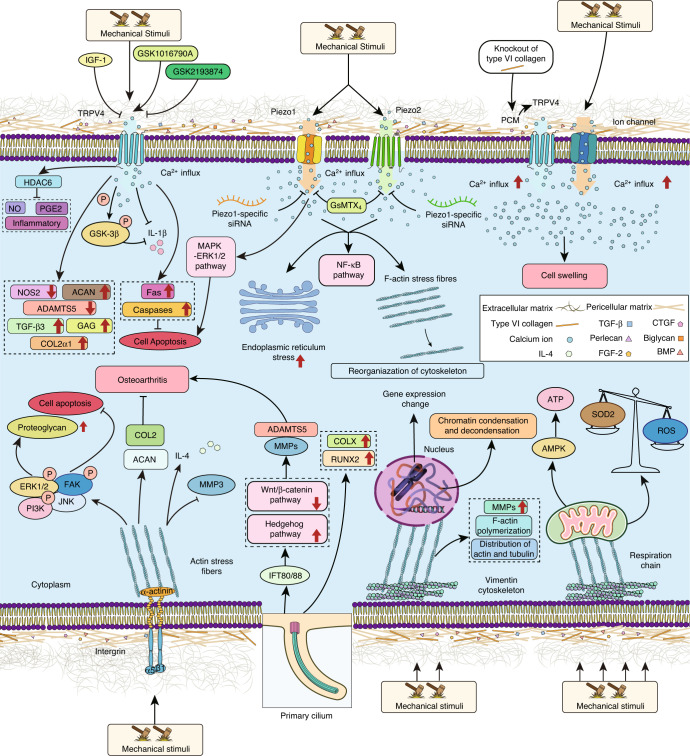
Different mechanosensors and their downstream pathways in articular chondrocytes. Pointed arrows indicate stimulatory effects, whereas blocked lines indicate inhibitory effects. IGF-1: insulin-like growth factor-1, TRPV4: transient receptor potential channel subfamily V member 4, GSK1016790A: TRPV4 agonist, GSK2193874: TRPV4 antagonist, GSK-3β: glycogen synthase kinase 3beta, HDAC6: histone deacetylase 6, PGE2: prostaglandin E2, NOS2: nitric oxide synthase, ACAN: aggrecan, ADAMT: a disintegrin and metalloproteinase with thrombospondin motifs, TGF-β: transforming growth factor-beta, GAG: glycosaminoglycan, IL: interleukin, NO: nitric oxide, COL2: type II collagen, GsMTX4: selective Piezo-inhibiting peptide, NF-κB: nuclear factor κB, F-actin: filamentous actin, MAPK: mitogen-activated protein kinase, ERK: extracellular signal-regulated kinase, PCM: pericellular matrix, CTGF: connective tissue growth factor, FGF: fibroblast growth factor, BMP: bone morphogenic protein, FAK: focal adhesion kinase, PI3K: phosphatidylinositol 3-kinase, JNK: c-Jun amino-terminal kinase, MMP: matrix metalloproteinase, IFT80/88: intraflagellar transport 80/88, COLX: type X collagen, RUNX2: Runt-related transcription factor 2, and AMPK: AMP-activated protein kinase

Mechanosensors respond to mechanical stimulation that alter the molecular/cellular structures to induce electrochemical and biological signals. Since mechanosensors are the initiators of signaling cascades, we summarize current mechanotransduction pathways based on mechanosensing effects (Fig. 2). In addition, the related mechanotransducers and mechanoimpactors in each pathway are discussed in the following sections. The functions of mechanotransduction-relevant molecules in maintaining cartilage health and promoting OA pathogenesis are detailed in Table 1.

**Table 1:** List of known mechanosensors in chondrocytes and their potential downstream genes and pathways

Mechanosensors in chondrocytes	Types of mechanical stimulation	Potential downstream molecules or pathways	Relationship with OA and cartilage health	Reference
TRPV4	N/A	N/A	The absence of TRPV4 resulted in osteoarthritic changes, particularly in male mice, suggesting a chondroprotective role for TRPV4 that is possibly mediated by its Ca^2+^ gating in response to hypotonicity.	^25^
	Mechanical (10% cyclic tensile strain (CTS), 0.33 Hz, 24 h) or osmotic loading (200, 315 or 400 mOsm, referred as hypo-, iso- or hyperosmotic, 24 h)	Inhibiting NO, PGE2, and inflammation through regulating HDAC6	Mechanical, osmotic, or pharmaceutical activation of TRPV4 regulates the HDAC6-dependent modulation of ciliary tubulin and is an anti-inflammatory factor in chondrocytes.	^26^
	Anterior cruciate-ligament transection (ALCT)	Activation of TRPV4 increases Ca^2+^ influx to trigger apoptosis by increasing FAS-associated protein with death domain and cleaved caspase-3, -6, -7, and -8 levels.	TRPV4 is upregulated in ALCT-induced OA articular cartilage, and administration of a TRPV4 inhibitor attenuates cartilage degeneration.	^121^
	Dynamic compressive load (a 10% peak-to-peak sinusoidal strain (7% offset) at 1 Hz for 3 h·d^−1^); hypo-osmotic treatments (400 → 200 mOsm and 600 → 400 mOsm, respectively)	Upregulated gene expression of TGFβ3, COL2a1, ACAN as well as increases in total GAG and collagen; downregulated ADAMTS5 and NOS2	TRPV4-dependent intracellular Ca^2+^ signaling has been detected in agarose-embedded chondrocytes. Inhibition of TRPV4 during dynamic loading prevents mechanically mediated regulation of proanabolic and anticatabolic genes.	^19^
	Natural aging; destabilization of the medial meniscus (DMM)	N/A	Loss of TRPV4-mediated cartilage mechanotransduction in adulthood reduces the severity of aging-associated OA. However, loss of chondrocyte TRPV4 does not prevent OA development following the destabilization of the medial meniscus (DMM).	^122^
	Viscoelasticity of alginate hydrogels mimic environment with different mechanical stress	TRPV4 controls the phosphorylation of GSK3β, which has been associated with changes in anabolic gene expression	The TRPV4-GSK3β molecular axis is disrupted in osteoarthritic chondrocytes; therefore OA cells cannot sense or respond to altered viscoelasticity of the surrounding matrix. Restoring chondrocyte ability may help slow the progression of OA.	^27^
Piezo1/2	Chondrocytes are probed via atomic force microscopy. Cartilage explants are injured with a 3 mm stainless steel punch that penetrates cartilage and reaches to bone	Ca^2+^ flux triggers chondrocyte apoptosis	High strain leads to Ca^2+^ flux into chondrocytes via PIEZO channels; and inhibiting Piezos with the peptide GsMTx4 protects articular chondrocytes from mechanically induced cell death.	^28^
	Cyclic stretching for 0 h/2 h/12 h/24 h/48 h at a 20% amplitude and a period of 10 times per minute	Upregulation of Piezo1 results in highly expressed Kif18A and β-tubulin, leading to chondrocyte cell cycle arrest and inhibits chondrocyte proliferation	Piezo1-siRNA reduces the inhibition of chondrocyte proliferation induced by mechanical stretch by downregulating the expression of Kif18A and inhibiting the depolymerization of microtubules.	^123^
	Static compressive loading for 0, 2, 12, 24, and 48 h	Activation of the MAPK/ERK1/2 signaling pathway	Piezo1 causes the apoptosis of the human chondrocyte by activating the classic MAPK/ERK1/2 signal pathway.	^32^
	High-speed pressure clamp and elastomeric pillar arrays	N/A	Both TRPV4 and PIEZO1 channels contribute to currents triggered by stimulation applied at cell–substrate contacts, but only PIEZO1 mediates stretching-activated currents in chondrocytes.	^29^
	AFM probe (flat, tipless) compresses single cells cyclically with a trigger force of 300–500 nN every 10 s for 2 min)	Piezo1 induction of excess intracellular Ca^2+^ at baseline, with elevated resting state Ca^2+^ in turn rarefying the F-actin cytoskeleton and amplifies mechanically induced deformation-based microtrauma	Increased Piezo1 expression in chondrocytes results in a feed-forward pathomechanism whereby the increased activity of Piezo1 induces excess intracellular Ca^2+^ at baseline and accelerates OA progression.	^30^
Primary cilium	Cyclic loading strain at 5%, 1 Hz on primary human chondrocytes	Primary cilia induction of ERK1/2 and CITED2 expression, leading to suppressed expression of MMP-1 and -13	Primary cilia sense moderate cyclic loading to inhibit the expression of MMPs and thus slow OA progression.	^36^
	N/A	Upregulation of Hedgehog (Hh) signaling (Ptch1 and Gli1), leading to overexpressed Mmp1, ADAMST5, ColX, Runx2	Deletion of IFT (intraflagellar transport) 88 can prevent primary cilium assembly in cartilage, which leads to acquisition of an OA phenotype characterized by reduced stiffness and increased expression levels of OA markers, including MMP-13, Adamts5, ColX, and Runx2 levels	^124^
	Indentation strain: A plane-ended impermeable cylinder (178 µm diameter) is applied to tissue with a tare load of 0.05 g and held for 200 s. Further indentation force is applied in increments of 5 µm with 200 s relaxation time between each application	N/A	Loss of primary cilia causes a significant reduction in the mechanical properties of cartilage, particularly in the deeper zones and possibly the underlying bone. This loss confirms the importance of primary cilia in the development of mechanically functional articular cartilage.	^125^
	N/A	Lack of primary cilia leading to reduced hedgehog (Hh) signaling and increased Wnt signaling	Loss of IFT80 and primary cilia block chondrocyte differentiation by disrupting ciliogenesis and altering Hh- and Wnt-mediated signal transduction, which in turn alters articular cartilage formation.	^126^
	Cyclic tensile strain (CTS; 0.33 Hz, 10% or 20% strain)	Hedgehog signaling	Mechanical loading activates primary cilium-mediated hedgehog signaling and ADAMTS-5 expression in adult articular chondrocytes, but this response is lost at high strain due to HDAC6-mediated cilia disassembly.	^33^
Integrin	N/A	Activate inflammatory and degradative mediators through Fyn, FAK	Activates αVβ3 signaling in many articular cell types and contributes to inflammation and joint destruction in OA.	^39^
	Knee joints are stressed either by forced exercise (moderate mechanical load) or by partial resection of meniscus followed by forced exercise (high mechanical load)	Lack of α5β1-Fibronectin binding induction of matrix-degrading enzymes, MMP3, MMP13	OA induced by meniscectomy followed by forced exercise accelerates cartilage degradation in α5β1 integrin-depleted mice.	^127^
	Pressure-induced strain (PIS) at a frequency of 0.33 Hz (2 s on/1 s off) for 20 min	Integrin-dependent signaling pathway leading to the opening of SK (small conductance Ca^2+^-dependent K^+^ channels) and membrane hyperpolarization by IL-4	Integrin-regulated production of IL-4 confers protection to chondrocytes by inhibiting cartilage degradation and promoting matrix synthesis in normal articular cartilage.	^44^
Pericellular matrix	Hypotonic (165 mOsm, ionic strength (IS) = 0.075 M), isotonic (330 mOsm, IS = 0.15 M), and hypertonic (550 mOsm, IS = 0.23 M) stress	Decorin, a small leucine-rich proteoglycan, is a key determinant of cartilage pericellular matrix micromechanics, with defective decorin exhibiting decreased intracellular calcium activity under both physiological and osmotically treated fluid environmental conditions in situ	Decorin is an essential constituent of the native cartilage matrix, and pericellular matrix of decorin-null murine cartilage leads to reduced content of aggrecan, the major chondroitin sulfate proteoglycan in cartilage, and a mild increase in collagen II-based fibril diameter, as well as a significant reduction in the cartilage micromodulus, suggesting that modulating decorin activities may enhance cartilage regeneration.	^128^
	Isotonic (300 mOsm) or hypotonic (200 mOsm) stress	Col6a1-knockout in PCM increases TRPV4-mediated calcium signaling and cell swelling	Alterations in PCM properties in OA or aging can influence mechanotransduction via TRPV4 or other ion channels, thereby influencing cartilage health.	^50^
Cytoskeleton	Continuous hydrostatic pressure (24 MPa) for 3 h	Actin and tubulin composed of cytoskeletal influence the number of cell organelles	Pressure induced OA-like distribution of actin and tubulin and a reduction in the number of cell organelles involved in the synthesis of collagen and proteoglycans.	^129^
	Homogeneous isotropic compression/stretch of 10% strain	N/A	Cytoskeletal Vimentin-disrupted chondrocytes showed a lower fluidization–resolidification response rate and reduced cellular stiffness.	^130^
	Continuous, uniaxial, and unconfined compressive load (0.5 MPa, Hz) for 10 and 30 min	Reduction in F-actin polymerization, resulting in increased expression of catabolic mediators (MMPs)	Thymosin β4, a putative mechanically regulated gene that inhibits F-actin polymerization results in increased expression of catabolic mediators (MMPs), leading to increased cartilage catabolism.	^131^
Nucleoskeleton	Hyperosmotic conditions (>400 mOsm·kg^−1^), hypo-osmotic conditions (100 mOsm·kg^−1^)	Nuclear morphology change induces chromatin condensation and decondensation, lead to altered gene expression	Alterations in chromatin structure are thought to influence gene expression and thereby regulate chondrocyte activity in response to mechanical stimulation, while the relation with OA needs further investigation.	^56^
	15% shear strain	Disruption of the nuclear envelope associated with lamin A/C depletion significantly increases nuclear strain in regions with low DNA concentration	Disruption to the nucleoskeleton induces an ellipsoidal morphology in nuclei and drives chondrocyte phenotype switching to fibroblast-like cells under load strain stimulation.	^57^
Mitochondria	Destabilization of the medial meniscus (DMM) induces abnormal loading in chondrocytes of knee cartilage	Mitochondrial superoxide, Sod2	Mechanical overload inhibits mitochondrial superoxide generation and Sod2 in cartilage, and its maintains continuous and accelerated mitochondrial oxidative damage in chondrocytes.	^65^
	A single impact load (a 500 gm weight dropped from a height of 50 mm)	Loading induces calcium release from the endoplasmic reticulum, causing mitochondrial membrane polarization and then activates caspase 9 to induce cell death.	Mitochondrial membrane depolarization via calcium quenching reduces the rate of impact-induced chondrocyte death.	^132^

The relationship between mechanosensors and OA is shown

*TRPV4* transient receptor potential channel subfamily V member 4, *NO* nitric oxide, *PGE2* prostaglandin e2, *HDAC6* histone deacetylase 6, *TGF-β* transforming growth factor-beta, *COL2* type II collagen, *ACAN* aggrecan, *GAG* glycosaminoglycan, *ADAMT* a disintegrin and metalloproteinase with thrombospondin motifs, *NOS2* nitric oxide synthase, *OA* osteoarthritis, *GSK-3β* glycogen synthase kinase 3beta, *GsMTX4* selective Piezo-inhibiting peptide, *MAPK* mitogen-activated protein kinase, *ERK* extracellular signal-regulated kinase, *AFM* atomic force microscopy, *CITED2* E/D-rich carboxy-terminal domain-2, *MMP* matrix metalloproteinase, *Hh signaling* Hedgehog signaling, *COLX* type X collagen, *RUNX2* Runt-related transcription Factor 2, *IFT80/88* intraflagellar transport 80/88, *FAK* focal adhesion kinase, *PCM* pericellular matrix, *F-actin* filamentous actin, *Sod2* superoxide dismutase 2, and *NA* not available

### Mechanically gated ion channels and porin-mediated pathways

Mechanically gated ion channels and porins are the most best characterized mechanosensors in chondrocytes. In general, these transmembrane proteins undergo structural changes upon mechanical loading and subsequently alter calcium flux. Calcium is second messenger that further influences intracellular signaling pathways.^23^

*Transient receptor potential cation channel subfamily V member 4 (TRPV4)*, a Ca^2+^-permeable osmomechano-TRP channel, is highly expressed in articular chondrocytes and mediates the cell response to mechanical loading.^24^ Activation of TRPV4, via moderate mechanical loading or selective chemicals, has been found to enhance anabolic and suppress catabolic gene expression in cultured chondrocytes.^19^ In contrast, inhibiting TRPV4 reduces the anabolic response to mechanical loading. Clark et al. discovered that *TRPV4*-deficient mice (*TRPV4*^−/−^) exhibited severe osteoarthritic changes.^25^ Interestingly, the role of TRPV4 was affected by sex and age. For example, male *TRPV4*^−/−^ mice presented with a greater calcified meniscal volume and cartilage damage than age-matched female mice. To investigate the mechanisms involved in these outcomes, Fu et al. used multiple stimuli, including mechanical loading (10% cyclic tensile strain, 0.33 Hz), osmotic stimulation, and chemical agonist stimulation to activate TRPV4 in primary chondrocytes and articular cartilage explants in vitro. They found that TRPV4 could inhibit interleukin (IL)-1β-mediated nitric oxide (NO) and prostaglandin E2 (PGE2) release and prevent cartilage degradation.^26^ The results strongly suggested that TRPV4 slows OA progression by inhibiting inflammatory pathways. However, contradictory results have also been reported. Specifically, Agarwal et al. found that treating chondrocytes with GSK101, a small-molecule agonist of TRPV4, increased the levels of intracellular Ca^2+^ and phosphorylated GSK3β, leading to a significant decrease in the gene expression of collagen type II and an increase in the levels of proinflammatory cytokines.^27^ Notably, the conclusion was drawn based on the use of a synthesized small molecule, which may not be an exclusive agonist for TRPV4. The potential influence of this inhibitor on other molecules may have confounded its effects on TRPV4 activation. Another possible reason for the discrepant findings, as reported in the same study, involves disruption to the TRPV4–GSK3β molecular axis in OA chondrocytes. In summary, it is likely that activating TRPV4 may impact normal and OA chondrocytes differently, a possibility that needs to be further investigated.

*Piezo1* and *Piezo2* are two important ion channels in chondrocytes. In contrast to TRPV4, which is primarily thought to play a protective role in cartilage, recent evidence has suggested that Piezo 1 and 2 are activated when injurious forces are applied. For example, a recent study by Lee et al. showed that activation of Piezo1 and Piezo2 after detrimental loading induced cartilage degradation and inflammation, and these effects were blocked by Piezo-specific blocking peptides and Piezo1- or Piezo2-siRNA.^28^ Servin-Vences et al. performed an RT‒qPCR analysis to determine the number of *Piezo1*, *Piezo2*, and *TRPV4* transcripts in chondrocytes and found high expression levels of *TRPV4* and *Piezo1* but not *Piezo2*. Interestingly, both TRPV4 and Piezo1 contribute to currents activated by stimulation applied at cell–substrate contact sites, but only Piezo1 mediated stretching-activated current.^29^ These studies suggested that the exact roles of Piezo1 in chondrocytes need to be further elucidated, and they also highlight the overlapping function of ion channels in transducing mechanical signals.

Furthermore, inflammatory cytokines, such as IL-1α, have been shown to promote the expression of *Piezo1*, which results in excessive steady-state calcium levels and hypersensitivity of chondrocytes to mechanical loading.^30^ Given the connection between activation of Piezo 1/2 and OA progression, cytokines serve as rational targets for developing disease-modifying drugs.

Although TRPV4 and Piezo1 represent the two most important ion channels involved in mechanotransduction, their interaction in chondrocytes is, surprisingly, not fully understood. Du et al. indicated that TRPV4-mediated calcium signaling plays a central role in the chondrocyte response to physiological strain levels (3% and 8%), while Piezo1/2-mediated calcium signaling was critical for injury caused by various strain levels (13%–18%).^31^ In the same study, knockout of *TRPV4* significantly suppressed stretching (3% strain)-induced calcium flux but did not influence the cell response to injurious stretching (>13% strain). Servin-Vences et al. reported similar results: depletion of TRPV4 did not affect the Piezo1-dependent response of murine chondrocytes to stretching.^29^ Therefore, even though these ion channels all function through calcium flux, they can act independently. In fact, in a study with human periodontal ligament cells (hPDLCs),^32^ Shen et al. found that extracellular signal-regulated kinase (ERK) participated in mechanotransduction mediated by the Piezo1 channel but not that mediated by TRPV4. Therefore, understanding the specific downstream pathways activated by these proteins is critical to tease out the different roles played by TRPV4 and Piezo1/2 in mediating the chondrocyte response to various mechanical stimuli.

### Cilia-mediated pathways

Cilia are microtubule-containing structures that extend from the cell surface into the extracellular space. They can directly participate in cell sensing of different stimuli, including physical force. Thompson et al. revealed that intact cilia activated the hedgehog signaling pathway and induced the expression of a disintegrin and metalloproteinase with thrombospondin motifs-5 (*ADAMTS-5*) under 10% cyclic tensile strain (CTS).^33^ However, this response was lost when the strain magnitude was increased up to 20% due to cilia disassembly due to excessive stretching of chondrocytes. Interestingly, the inhibition of histone deacetylase 6 (HDAC6) prevented cilia disassembly and restored mechanosensitive hedgehog pathway function under 20% CTS.^33^ Moreover, chondrocytes in OA cartilage carry cilia with different characteristics than chondrocytes om healthy cartilage.^34^ For example, interleukin-1-treated primary chondrocytes exhibited a 50% increase in cilia length after 3 h of exposure.^35^ However, how this elongation of cilia affects the chondrocyte response to force needs to be further investigated.

ED-rich tail 2 (CITED2) is another target that is regulated by primary cilia when chondrocytes are subjected to load strain. Abolishing primary cilia by knockdown of intraflagellar transport protein (IFT88) blocked the transactivation of CITED2 and upregulated the expression of metalloproteinase (MMP)-1 and MMP-13 (ref. ^36^). Juhász et al. applied a uniaxial intermittent cyclic load to generate hydrostatic pressure and fluid shear stress on chondroprogenitor cells.^37^ The results showed that the activity of protein kinase A (PKA) was enhanced along with its downstream transcription factor CREB, together activating the chondrogenic transcription factor Sox9 to promote the generation of collagen II and other extracellular matrix material.

In summary, primary cilia function as mechanosensors, and their activity is mediated through two mechanisms, namely, as classical cell sensors of fluid flow and pressure^38^ and modulators of the PKA pathway, which depends on primary cilia length in chondrocytes.^35^ Moderate mechanical stimuli activate primary cilia and promote the secretion of extracellular matrix components, while excessive loading can induce cilia disassembly and result in injuries to cells.

### Integrins and ECM-mediated pathways

Integrin family members are widely expressed in articular chondrocytes as important cell adhesion molecules. Various types of integrins are expressed in chondrocytes, including α1β1, α3β1, α5β1, α10β1, αVβ1, αVβ3, and αVβ5.^39–41^ They play critical roles in the interactions between chondrocytes and the ECM. Integrins are mechanosensors in various cell types and are present in chondrocyte cilia. Zhou et al. incubated C-28/I2 and C-20/A4 human chondrocyte cell lines with an anti-β(1) integrin (CD29) function-blocking antibody and then applied mechanical stimulation to the monolayer cell culture. The results showed that the mechanical stimulus activated Jun N-terminal kinase (JNK) and increased proteoglycan synthesis, which was partially dependent on integrin.^42^ Another study proved that hydrostatic compressive forces (HCFs) significantly increased the expression levels of integrin α2, integrin α5, and integrin β1 and thus increased the phosphorylation levels of FAK, ERK1/2, and PI3K in a dose-dependent manner.^43^ It also revealed that HCFs enhanced the viability of chondrocytes by inhibiting apoptosis through the integrin-FAK-ERK/PI3K pathway. Additionally, previous studies had demonstrated that integrin-mediated chondrocyte responses to mechanical stimulation promoted the secretion of interleukin 4 (IL-4), which resulted in membrane hyperpolarization.^44^ Studies of cytokine effects on chondrocytes in vitro have suggested that IL-4 alters the ratios of MMP to TIMP, favoring TIMPs by suppressing IL-1–stimulated MMP3 production.^45,46^ This chondroprotective response is absent in chondrocytes from OA cartilage.^47^ Activation of integrins by mechanical loading has also been shown to elevate the gene expression of aggrecan and decrease the expression of MMP-3. Interestingly, these changes are blocked by anti-IL-4 antibodies, highlighting the chondroprotective role of IL-4 in connecting integrins and mechanoimpactors.^47^

In hyaline cartilage, chondrocytes are surrounded by a dense extracellular matrix. Based on the distance to the chondrocyte membrane, cartilage ECM is divided into three parts: the pericellular, interterritorial, and territorial matrices. Specifically, the pericellular matrix, which is primarily composed of collagens (e.g., VI, IX, X, and XI), noncollagenous proteins, and proteoglycans,^48^ forms the immediate microenvironment of chondrocytes. Since all physical forces influence cells only through the ECM, ECM components are considered to be mechanosensors.^49^ However, the downstream targets of these ECM-mediated effects of force in cells are not clear. Given that the ECM mostly functions primarily through binding integrins, the deformation of the ECM may alter ECM component interactions with integrins, thereby changing integrin-relevant pathways. In addition, ECM components may regulate the level and state of the ion channels described above. For instance, Zelenski et al. showed that knockout of type VI collagen increased chondrocyte swelling, which may be mediated by elevating osmosis-induced TRPV4 signaling.^50^

In summary, the integrin family plays a critical role in mediating interactions between cells and the extracellular matrix, but the precise function of each integrin as a mechanosensor in chondrocytes has not been fully elucidated. ECM components can couple with multiple exoproteins, such as integrin and ion channels, to form conjugated mechanosensing complexes. Decoding the pathways involved in their conjugation and the mechanosensing modes of these complexes is essential for understanding the behaviors of chondrocytes under mechanical stimulation. Notably, the microfibril alignment and composition in the ECM are variable extensively in different cartilage layers.^51^ For example, the superficial layer contains a relatively small amount of proteoglycan, and the fibers are arranged parallel to the joint surface.^51^ Farther into the articular cartilage, the collagen fibers are oriented perpendicular to the joint surface. Moreover, collagen fibrils in the middle layer exhibit a larger diameter than those in the superficial layer, with a higher concentration of proteoglycans and lower amounts of water and collagen in the former. In the deep zone, collagen fibrils with the largest diameter, the highest concentration of proteoglycans, and the lowest concentration of water are found. The biomechanics of articular cartilage leverage these microanatomical features to reduce the forces of friction across the joint to extremely low values.^51^ We believe that these structural and compositional differences in the ECM in different zones influence the mechanical response of resident chondrocytes, which deserves further investigation.

### Cytoskeleton- and nucleoskeleton-mediated pathways

Since cells are not rigid, mechanical loading of sufficient intensity can directly cause cell deformation, which results in dynamic changes to the cytoskeletal and nucleoskeletal structure.^52^ The cytoskeleton is a complex three-dimensional network consisting of cell microtubules, microfilaments, and intermediate fibers. The nucleoskeleton is also a three-dimensional network structure within the nuclear envelope, and it includes the nuclear matrix, nuclear lamina and pore complex, and chromosomal skeleton. Notably, the cytoskeleton is structurally integrated with the nucleoskeleton, and these integrated areas are called linker of the nucleus to cytoskeleton (LINC) complexes, and they enable the synergistic effects induced by mechanical stimuli.^53^ Notably, a dynamic change in skeleton structures can facilitate the movement of intracellular organelles, such as cell nuclei and mitochondria,^54,55^ and further influence chondrocyte DNA concentration and gene expression through signalling mediated by the nucleoskeleton.^56,57^ Reynolds et al. found that the disruption of the nuclear envelope associated with lamin A/C depletion significantly increases nuclear strain in regions of low DNA concentration. The increased nuclear peak strain shifts the phenotype of chondrocytes to a fibroblast-like phenotype by increasing the contractility of the actin cytoskeleton.^57^

In addition to the mechanosensing and mechanotransduction roles of the cytoskeleton, mechanical loading can alter the cytoskeletal structure. In applying dynamic osmotic loading to chondrocytes and using phalloidin staining to observe changes to the actin cytoskeleton, Chao et al. found that dynamic osmotic loading resulted in a more uniformly distributed actin cytoskeleton.^58^ Erickson et al. showed that osmotic stress-induced transient increases in Ca^2+^ levels and simultaneously caused a reorganization of intracellular actin through a mechanism that required Ca^2+^ in the extracellular medium.^59^ The results also indicated that cytoskeletal reorganization was partially mediated by Ca^2+^ flux. Overall, there is consensus indicating that the cytoskeleton and nucleoskeleton are vital structures through which cells sense mechanical stimuli, but the precise effects exhibited by these elements in diverse biological activities have not been explored.

In summary, mechanical loading on the cytoskeletal structure not only can influence organelles but can also directly affect cytoskeleton-associated pathways and molecules. For example, previous research revealed that mechanical force-related actin depolymerization “frees up” ROCK to mediate an increase in Col2 expression.^60^ Zhen et al. found that mechanical overload (via cellular contractile forces >40 pN) activated TGFβ mediated through actin filaments, and overexpressed TGFβ induced cartilage degeneration in OA.^41^ In the future, researchers should continue to investigate these mechanosensitive cytoskeleton-associated pathways and molecules and clarify their roles in the progression of OA.

### Mitochondria

Previous research has suggested that mitochondria act as mechanosensors and impact a series of downstream signaling pathways by mediating cellular responses to mechanical loading.^61,62^ Through mitochondrial links to the cytoskeleton, mechanical loading may directly cause mitochondrial deformation. An instance of high kinetic energy produced by mechanical loading induced chondrocyte injury and caused acute mitochondrial dysfunction and altered cellular respiration. Sauter et al. applied two agents (cytochalasin B and nocodazole) that promoted the dissolution of microfilaments and microtubules in chondrocytes and found that both agents significantly reduced the impact-induced oxidant release from mitochondria, thereby preventing cell death.^63^ In accordance with previous results, Bartell et al. tracked the structural changes in mitochondria after mechanical impact by using electron microscopy to reveal mitochondrial loss of fission and cristae structures within a few minutes of mechanical injury.^64^ The Szeto–Schiller (SS) peptide, a newly developed mitochondrial-protecting drug that selectively targets the inner mitochondrial membrane, has been shown to protect the mitochondrial structure and preserve mitochondrial function in chondrocytes that had been subjected to mechanical overload.^64^ These results strongly indicate that mitochondria are important mechanosensors and play essential roles in the mitochondrial-cytoskeletal linkage that is involved in mechanically induced cartilage degeneration.

Respiratory chain complexes are parts of the mitochondrial biophysical structure, which plays a central role in energy metabolism and adenosine triphosphate (ATP) production. Moreover, the respiratory chain is also the primary source of superoxide radicals in various cell types, including chondrocytes. Through dynamic loading of bovine osteochondral explants, Wolff et al. revealed that physiological loading stimulated ATP production and the release of reactive oxygen species (ROS) and that these effects were significantly suppressed by the mitochondrial ROS scavenger MitoQ.^54^ A small amount of ROS generated under physiological loading are involved in intracellular signaling, which promote the phosphorylation of AMP-activated protein kinase (AMPK)to regulate chondrocyte metabolic homeostasis.^61^ The processes that produce and eliminate ROS in cells are maintained in dynamic equilibrium. For example, superoxide dismutase 2 (SOD2) metabolizes ROS in mitochondria and attenuates their harmful effects in cells. However, excessive mechanical loading impairs respiratory chain complex function, leading to the release of large amounts of ROS from mitochondria and downregulated SOD2, causing the breakdown of the SOD2-centric oxidation‒reduction balance and leading to chondrocyte apoptosis and cartilage degeneration.^65,66^

In recent studies, considerable attention has been focused on mitochondrial dysfunction induced by mechanical loading in OA, but the underlying mechanisms are often overlooked. Importantly, as highly dynamic organelles, mitochondria remain functional via the balance of fission and fusion. Mitochondrial biogenesis and mitophagy are two additional processes that contribute to their dynamic balance.^61^ Investigating the effects of mechanical stimulation on these mitochondrion-related processes is important for understanding the mechanosensing role of mitochondria in chondrocytes.

## Transcription factors in mechanotransduction pathways in chondrocytes

**Fig. 3:**
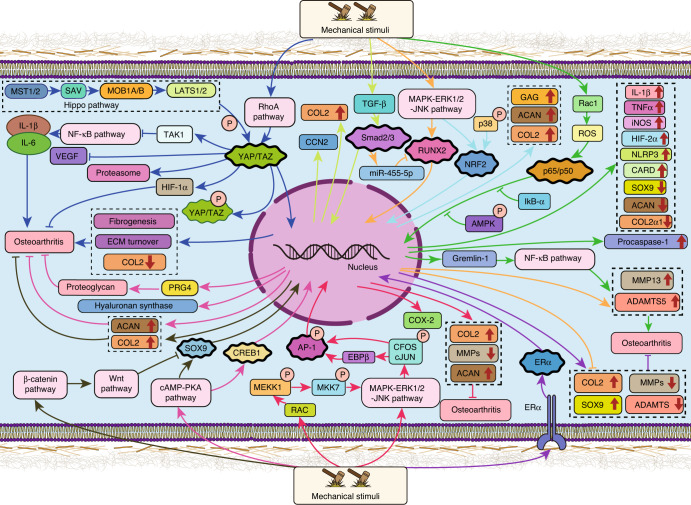
Representative transcription factors that mediate chondrocyte responses to mechanical stimulation. Different colors indicate different pathways. Red indicates a supportive effect, pointed arrows indicate stimulatory effects, and blocked lines indicate inhibitory effects. YAP: Yes-Associated Protein, SAV: salvador, MST1/2: serine/threonine kinases STK4 (MST1) and STK3 (MST2), LATS1/2: large tumor suppressor 1 and 2, MOB1 A/B: MOB kinase activator 1, RhoA: Ras homolog gene family member A, TAZ: transcriptional coactivator with PDZ-binding motif, TAK1: Tgf-β-activated kinase1, IL1β: interleukin-1β, VEGF: vascular endothelial growth factor, HIF-1α: hypoxia-inducible factor 1-α, COL2: collagen type II, PRG4: human proteoglycan 4, ACAN: aggrecan, SOX9: SRY-related high-mobility-group-box gene 9, CREB1: cAMP-response element-binding protein, CCN2: cellular communication network factor 2, TGF-β: transforming growth factor-β, Smad2/3: SMAD family member 2/3, RUNX2: Runt-related transcription factor 2, NRF2: nuclear factor erythroid2-related factor 2, AMPK: AMP-activated protein kinase, IkB-α: inhibitor of NF-κB-α, ROS: reactive oxygen species, Rac-1: Ras-related C3 botulinum toxin substrate 1 (Rac1), GAG: glycosaminoglycan, TNFα: tumor necrosis factor α, iNOS: inducible nitric oxide synthase, HIF-2α: hypoxia-inducible factor-2α, NLRP3: nucleotide-binding oligomerization domain, leucine-rich repeat and pyrin domain-containing 3, CARD: caspase raise structural domain, MMP13: matrix metalloproteinase 13, ADAMT5: a disintegrin and metalloproteinase with thrombospondin motifs-5 (ADAMT-5), EBPβ: CCATT enhancer-binding protein β (C/EBPβ), AP-1: activator protein 1, JNK: c-Jun N-terminal kinase, ERK: extracellular signal-regulated kinase, MAPK: mitogen-activated protein kinase, MKK7: mitogen-activated protein kinase kinase 7

Transcription factors (TFs) are proteins that bind to the cis-acting promoter element to convert or transcribe DNA into RNA, thus playing important roles in transducing the signals from mechanosensors into cell nuclei, in turn amplifying the effects of mechanical stimulation. In Fig. 3, we summarize representative transcription factors that are known to mediate chondrocyte responses to mechanical stimuli. Their functions are detailed in Table 2. Notably, the molecules listed as mechanosensitive TFs also respond to other factors or cellular activities. To the best of our knowledge, there is no TF that is exclusively activated by mechanical stimulation.

**Table 2:** Mechanosensitive transcription factors (TFs) in chondrocytes

Mechanosensitive TFs in chondrocytes	Cell, tissue, or animal model applications	Types of mechanical stimulation	The responsiveness of TFs to mechanical stimulation	Potential upstream pathways or mechanosensors	Downstream target genes or pathways	Relationship with OA or cartilage health	Reference
RelA/p65/p50	Mouse primary chondrocytes, surgically induced knee OA mouse model, and human articular cartilage surgical specimens	0.5 Hz, 10% cyclic tensile strain loading for 30 min	RelA/p65 activated by excessive mechanical loading	Ras-related C3 botulinum toxin substrate 1 (Rac1) enhances ROS production and activates p65.	Gremlin-1 and NF-κB induce catabolic enzymes and antagonize the induction of SOX9, COL2a1, ACAN.	The Rac1–ROS–NF-κB axis plays an essential role in Gremlin-1 induction by excessive mechanical loading, and Gremlin-1 activates NF-κB signaling, which results in the induction of catabolic enzymes such as MMP13 and ADAMTs5.	^4^
	Rabbit primary chondrocytes	Cyclic tensile strain at low magnitude (4%–8% equibiaxial strain) and high magnitude (15%–18% equibiaxial strain).	CTS at low magnitude inhibits nuclear translocation of p65 and p50; CTS at high magnitude induces rapid nuclear translocation of P65/P50	NA	Interleukin-1β (IL-1β), tumor necrosis factor α (TNFα), inducible nitric oxide synthase (iNOS), and matrix metalloproteinases (MMPs).	Nuclear translocation of NF-κB subunits p65 and p50 leads to the expression of proinflammatory genes and the synthesis of mediators of tissue destruction to promote OA.	^133^
	Chondrocytes isolated from the condylar cartilage of 3-week-old male Sprague‒Dawley (SD) rats.	Cyclic compressive force (CCF) at 0–4 000 μ-strain (μst) of 0.5 Hz for 2 h.	Mechanical stress significantly increases the expression of p65	N/A	Regulating HIF-2α and its downstream factors (MMP13 and ADAMTs-4).	Inhibition of p65 *suppresses* HIF-2α and the expression of MMP13, ADAMTs4, and slows temporomandibular joint OA.	^134^
	SD OA rats; chondrocytes are obtained from the articular cartilage of knee joints of male SD rats.	The rats are exercised at low intensity for 30 min once per day, moderate intensity for 60 min once per day and high intensity for 90 min once per day (19.3 m·min^−1^ with 5° of inclination for 5 days per week for 4 weeks for all treadmill exercises); chondrocytes underwent cyclic tensile strain (10%, 0.5 Hz) for different durations (0, 0.5, 1, 2, 4, 8, and 16 h).	CTS for 4 h activates AMP-activated protein kinase (AMPK) phosphorylation and suppresses nuclear translocation of nuclear factor (NF)-κB p65	AMP-activated protein kinase (AMPK) phosphorylation suppresses the nuclear translocation of nuclear factor (NF)-κB p65.	Increases in proinflammatory cytokines, TNF-α and IL-1β, as well as ROS levels.	Moderate biomechanical stress reduces sensitization to the inflammatory response in articular cartilage and chondrocytes by inhibiting AMPK/NF-κB signaling pathway.	^77^
	MIA-induced OA rats; Primary rat chondrocytes.	The rats are exercised at 12 m·min^−1^ for 45 min once per day for 5 days per week for 4 weeks; chondrocytes are subjected to uniform biaxial strain (10%, 0.5 Hz) for 0, 1, 2, 4, 8, or 12 h.	Moderate-intensity treadmill exercise and moderate CTS inhibit the nuclear translocation of NF-κB p65	Cyclic tensile strain reduces the levels of ROS and inhibits NF-κB p65 nuclear translocation via the activation of IκB-α.	Stimulating the formation of the NLRP3 inflammasome leads to the production of proinflammatory cytokines, adaptor apoptosis-associated speck-like protein-containing a CARD, and procaspase-1.	Moderate cyclic tensile strain inhibits the activation of the NF-κB/NLRP3 signaling pathway and blocks chondrocyte destruction caused by TRAIL, presumably by abrogating IL-1β production and collagen II breakdown in chondrocytes.	^78^
YAP/TAZ	Rat cartilage chondrocytes; rat model of anterior cruciate ligament (ACL) transection surgery-induced knee instability.	Cyclic mechanical stress from 0 to 5 500 μ strain at 1 Hz in vitro; treadmill exercise in vivo.	Cyclic mechanical stress promotes YAP proteins expression in a magnitude-dependent manner. Cyclic mechanical stress at 4 000 μ strain exhibited the most significant effect to upregulate YAP.	N/A	Promoting HIF-1α stabilization and activation, as well as VEGF expression.	YAP activates HIF-1α, and HIF-1α promotes chondrocytes proliferation and regulates chondrogenic differentiation to suppress OA progression.	^135^
	Mouse model of anterior cruciate ligament transection (ACLT) surgery-induced knee OA.	N/A	YAP expression level clearly correlates with the severity of cartilage degradation in mice with surgery-induced OA.	Hippo signaling cascade consisting of MST1/2, SAV, LATS1/2, and MOB1A/B.	Inhibits inflammatory cytokines production, NF-κB signaling and proteasome-mediated degradation.	YAP is necessary and sufficient to attenuate OA progression by inhibiting inflammatory responses triggered by NF-κB signaling. MST and LATS kinases phosphorylate YAP/TAZ, and inhibit its transcriptional activity.	^136^
Runx2	SW1353 chondrocyte-like cells.	Uni-axial cyclic tensile strain (CTS) (0.5 Hz, 10% stretch) is applied for 30 min.	CTS induces the upregulated expression of RUNX-2.	Mitogen-activated protein kinase (MAPK) pathways.	MMP-13 and ADAMTS-5.	RUNX-2 mediates mechanical stress-induced MMP-13 and ADAMTS-5 expression to promote OA.	^137^
	Temporomandibular joint (TMJ) loading in rats.	Hard diet is fed to simulate physiological temporomandibular joint (TMJ) loading; soft diet is fed to reduce TMJ load.	Functional cartilage loading markedly increases the protein level of Runx2.	JNK and ERK MAPK cascades.	RUNX-2 can interact with other transcription factors, such as the SMADs activated by members of the bone morphogenetic protein (BMP) family.	RUNX-2 plays a critical role in the progression of chondrocyte maturation but is also normally expressed in late hypertrophic chondrocytes.	^138^
	Primary chondrocytes isolated from discarded endplate cartilage tissue of patients.	Mechanical tension 10% elongation with 0.5 Hz intermittent cyclic tension (8 h daily).	The expression of RUNX2 in human chondrocytes is significantly increased after tension loading.	TGF-β/SMAD signaling pathway upregulates miR-455-5p expression and thus reduces RUNX2 levels.	RUNX2 inhibits COL2 and SOX9 but stimulates MMP13 and COL10.	RUNX2 aggravates the tension-induced extracellular matrix loss in endplate cartilage.	^139^
Creb	Chondroprogenitor cells isolated from limb buds of 4-day-old chicken embryos.	Uniaxial intermittent cyclic load is transmitted in the culture medium as hydrostatic pressure and fluid shear (0.05 Hz, 600 Pa for 30 min).	Creb phosphorylation levels are increased and nuclear signal intensity is increased in response to mechanical stimulation.	Increases cAMP levels, significantly enhances protein kinase A (PKA) activity activated CREB and Sox9.	CREB and SOX9 elevate mRNA expression of several cartilage matrix constituents, including collagen type II and aggrecan core protein, as well as matrix-producing hyaluronan synthases.	PKA/CREB-SOX9 significantly augments cartilage matrix production.	^37^
	Mice primary chondrocytes from the superficial zone.	Cyclic tensile strain (0.5 Hz, 15% elongation) and fluid flow shear stress (180 rotations per minute) are applied in vitro; experimental OA mice model for in vivo experiments.	Mechanical loading increases the mRNA levels of Creb1	Activates the Wnt/β-catenin signaling pathway.	CREB1 enhances PRG4 expression.	PRG4 expression is enhanced, and cartilage degeneration is suppressed.	^82^
AP-1	Primary chondrocytes isolated from bovine.	Uniaxial, cyclic compression (1 kPa, 1 Hz, 30 min)	Mechanical stimulation increases AP-1 binding to DNA	Mechanical loading significantly increases the phosphorylation of both ERK1/2 and JNK, and then cFOS and cJUN are phosphorylated to induce AP-1 binding with DNA.	Inhibition of AP-1 prevents the upregulation of aggrecan and COL2a1, as well as MMP-3 and MMP-13 expression.	Blocking AP-1 binding prevents increases in matrix synthesis and accumulation, which promote cartilage formation.	^140^
	Human T/C-28a2 chondrocyte line	Shear stress is applied by using a parallel-plate flow chamber with a recirculating flow loop of 5 dyn per cm^2^ or that plateaus at 20 dyn per cm^2^	Shear stress induces the cis-response element, and AP-1 binds to the promoter region of Cox-2	Activates the Rac/MEKK1/MKK7/JNK2/c-Jun/C/EBPβ-dependent signaling pathway.	Upregulates the proinflammatory enzyme Cyclooxygenase-2 (COX-2).	Induces the expression of COX-2, which is involved in the pathogenesis and progression of arthritic disorders.	^141^
	Chondrocytes are isolated from cartilage tissue harvested from *6- to 9-month-old* bovine.	Cyclic compression (30 min, 1 kPa, 1 Hz)	Cyclic compression induces AP-1–DNA binding	N/A	Blocking AP-1–DNA binding prevents an increase in the transcription of MMP-3 and MMP-13 as well as increases in the accumulation of collagens and proteoglycans.	AP-1 can be activated by mechanical loading to promote *cartilage* matrix remodeling and increase extracellular matrix catabolic change.	^142^
Smad2/3	SW1353 human chondrocytes	Uni-axial cyclic stretching tensile strain (CTS, 0.5 Hz, 5% strain) is applied.	Uni-axial CTS induces the nuclear translocation of Smad2/3.	CTS may induce TGF-β1 release and stimulate Smad2/3 activity.	SMAD2/3 interact with the CCN2 promoter and the COL2A1 enhancer.	Physiological mechanical stress stimulates the induction of chondrogenic genes by increasing the formation of complexes composed of phosphorylated SMAD2/3 and SOX9.	^143^
Nrf2	Porcine chondrocytes are used to establish a 3-dimensional pellet model	The pellets are exposed to focused-model shockwaves applied in a custom-made acrylic thermostatic container with a membrane connecting to the shockwave applicator.	The phosphorylation of ERK1/2 and p38 and the nuclear translocation of Nrf2 are activated by shockwaves.	Transient ROS production, phosphorylation of ERK1/2 and p38 activate the nuclear translocation of Nrf2 are induced.	Nrf2 increases the gene expression of COL2a1 and ACAN, as well as promotes GAG synthesis.	Nrf2 inhibition reduces shockwave-enhanced ECM synthesis.	^144^
Sox9	Primary chondrocytes isolated from bovine calves	Physiological tensile strain (7.5% elongation, 1 Hz, 30 min) is applied.	Physiological tensile strain induces SOX9 transcription and expression.	Activation of Wnt signaling via β-catenin nuclear translocation blocks the physiological SOX9-stimulating effects of tensile strain.	SOX9 enhances chondrocyte phenotype acquisition to increase type II collagen (COL2a1) and aggrecan (ACAN) levels.	Pretreatment with Wnt3A represses Sox9 and the transcription of chondrocyte matrix genes, while enhancing the expression of catabolic mediators.	^145^

Types of mechanical stimuli, cells, tissues, and animal models used in all previous research are listed in the table; potential upstream mechanosensors or pathways and downstream targeted molecules or pathways of each TF are concluded

*TFs* transcription factors, *RelA* relative v-rel reticuloendotheliosis viral oncogene homolog A, *OA* osteoarthritis, *RAC-1* Ras-related C3 botulinum toxin substrate 1, *NF-κB* nuclear factor kappa-B, *Sox9* SRY-related high-mobility-group-box gene 9, *Col2a1* collagen, type II, alpha 1, *ACAN* aggrecan, *MMP13* matrix metalloproteinase 13, *ADAMT5* a disintegrin and metalloproteinase with thrombospondin motifs 5, *IL-1β* interleukin 1β, *TNFα* tumor necrosis factor α, *iNOS* inducible nitric oxide synthase, *CCFs* cyclic compressive forces, *HIF-1α* hypoxia-inducible factor 2-α, *AMPK* AMP-activated protein kinase, *ROS* reactive oxygen species, *IkB-α* inhibitor of NF-κB-α, *MIA* monosodium iodoacetate, *NLRP3* nucleotide-binding oligomerization domain, leucine-rich repeat and pyrin domain-containing 3, *CARD* caspase raise structural domain, *TRAIL* TNF-related apoptosis-inducing ligand, *YAP* Yes-Associated protein, *TAZ* transcriptional coactivator with PDZ-binding motif, *ACL* anterior cruciate ligaments, *HIF-1α* hypoxia-inducible factor 1-α, *MST1/2* serine/threonine kinases STK4 (MST1) and STK3 (MST2), *SAV* salvador, *LATS1/2* large tumor suppressor 1 and 2, *MOB1 A/B* MOB kinase activator 1, *CTS* cyclic tensile strain, *RUNX2* Runt-related transcription factor 2, *MMP13* matrix metalloproteinase 13, *TMJ* temporomandibular joint, *JNK* c-Jun N-terminal kinase, *ERK* extracellular signal-regulated kinase, *MAPK* mitogen-activated protein kinase, *BMP* bone morphogenetic protein, *TGF-β* transforming growth factor-β, *COL2* collagen type II, *COL10* collagen X, *PKA* protein kinase A, *CREB* cAMP-response element-binding protein, *PRG4* human proteoglycan 4, *AP-1* activator protein 1, *COX2* cyclooxygenase-2, *CCN2* cellular communication network factor 2, *NRF2* nuclear factor erythroid2-related factor 2, *GAG* glycosaminoglycan, *ECM* extracellular matrix, and *NA* not available

### Nuclear factor kappa-light-chain-enhancer of activated B cells (NF-κB)

The nuclear factor kappa-light-chain-enhancer of activated B cells (NF-κB) family includes five members: RelA (p65), RelB, c-Rel, NF-κB1 (p50), and NF-κB2 (p52).^67^ The most common form of NF-κB is a heterodimer of the p50 and p65/RelA proteins, which can activate the expression of TNF-α, IL-1β, IL-6, iNOS, COX2, chemokines, and adhesion molecules, among other proteins, and participate in inflammatory and immune responses in joints.^68^ In its inactivated state, the heterodimer comprising p50 and p65 usually combines with inhibitors of NF-κB (IκB). IκB can be phosphorylated by inhibitor of kappa B kinase (IκK) and then degraded via proteases to release the heterodimer of NF-κB, which plays roles with TFs. NF-κB has been identified as a mediator of mechanotransduction in several cell types. For example, an increase in the translocation of activated NF-κB to the nucleus has been observed in a number of cell types under mechanical stimuli, including chondrocytes,^69^ osteoblasts,^70^ and vascular endothelial cells.^71^ Chang et al. revealed that ras-related C3 botulinum toxin substrate 1 (Rac1) was overexpressed under excessive mechanical loading to promote the generation of ROS. Then, ROS phosphorylated IκBα (dual Ser32/36) to release the p65/p50 dimer, thus leading to joint inflammation and cartilage degradation.^4^ The activation of Rac1 has been shown to associate with alpha6/beta4 integrin.^72^ Changes in the formation or dissolution of actin filaments and microtubules can also affect the nuclear localization of NF-κB. In particular, the nuclear localization of activated NF-κB is correlated with the microtubule-based motor protein dynein.^73^ Mikenberg et al. used colchicine and vincristine to disrupt cellular microtubules and found ectopic accumulation of NF-κB in the nucleus and reduced NF-κB-dependent transcriptional activity.^74^ Moreover, the treatment of cells with low concentrations of TN16 (25 and 50 nmol·L^−1^), which suppressed microtubule dynamics without visibly affecting microtubule organization, enhanced the association of NF-κB with microtubules and facilitated the nuclear translocation of NF-κB.^75,76^ Interestingly, moderate mechanical stimulation inhibited the nuclear translocation of NF-κB and maintained cartilage health. For example, Yang et al. showed that cyclic tensile strain alleviated the chondrocyte damage induced by IL-1β by activating AMP-activated protein kinase (AMPK) phosphorylation and suppressing the nuclear translocation of nuclear factor (NF)-κB p65 (ref. ^77^). Additionally, moderate-intensity treadmill exercise inhibited the nuclear translocation of NF-κB p65 and the formation of nucleotide-binding and oligomerization domain-like receptor-containing protein 3 (NLRP3) by downregulating tumor necrosis factor-related apoptosis-induced ligand (TRAIL).^78^

### Yes-associated protein (YAP)

Previous research has strongly suggested that YAP is a downstream molecule of the ion channel protein Piezo1, and it plays crucial mechanotransduction functions in Piezo1-activated pathways.^15,16^ However, few studies have reported on the relationship between YAP and ion channel proteins in chondrocytes. Xie et al. embedded chondrocytes into a biomaterial scaffold to create a cartilage-like hydrogel and exposed this construct to mechanical stimuli. The results showed that the expression levels of YAP, Piezo1, and TRPV4 increased with increasing dynamic loading time.^79^ Another study reported that G protein-coupled estrogen receptor (GPER) promoted the expression and nuclear localization of YAP under mechanical stress, but it inversely inhibited the expression of Piezo1 in chondrocytes.^80^ The same study also showed that YAP was significantly decreased in degenerated cartilage, and silencing YAP caused chondrocyte apoptosis. However, contradictory results of YAP function in OA were reported in other studies. Zhang et al. constructed YAP conditional knockout (cKO) mice and found that cKO-YAP mice presented with an unchanged level of collagen type II and with cartilage that had been protected from degeneration in an OA model. Furthermore, intra-articular injection of the YAP-selective inhibitor verteporfin maintained cartilage homeostasis to a significant level in a mouse OA model.^81^ Therefore, the specific role and functions of YAP in chondrocytes exposed to mechanical stimuli still need to be further investigated.

### Other TFs function as mechanotransducers

Various TFs can function as mechanotransducers. For instance, mechanical loading upregulated Creb1 by increasing the cAMP level or activating WNT/β-catenin signaling. Mechanical stimulation of chondroprogenitor cells resulted in increased cAMP levels, significantly enhanced protein kinase A (PKA) activity and increased signal intensity of nuclear CREB, the archetypal downstream transcription factor mediating PKA signaling.^37^ By generating Prg4-Cre^ERT2^;Ctnnb1^fl/fl^ and Prg4-Cre^ERT2^;Ctnnb1-ex3^fl/wt^ mice with loss- and gain-of-function Wnt/β-catenin signaling in the cartilage superficial zone (SFZ), Xuan et al. revealed that OA development was significantly accelerated, and this increased rate was accompanied by decreased Prg4 expression and SFZ destruction in SFZ-specific β-catenin-knockout mice.^82^ Recently, our research team revealed that estrogen receptor α (ERα) is another TF that can function as a mechanotransducer in chondrocytes by mediating the expression of various OA-related genes.^11^ For example, damaging mechanical loads inhibited the expression of ERα, which in turn downregulated chondrogenic genes (*COL2* and *SOX9*) and upregulated degenerative genes (*MMP13* and *ADAMTS4*). When ERα was eliminated, mechanical loading continuously induced ECM turnover and led to cartilage ossification. Moreover, as described above, ERα participates in mechanotransduction in other cell types. Therefore, studies on ERα to date provide a solid starting point to further elucidate the roles played by ERα in mediating chondrocyte responses to various forces.

With the development of large-scale gene sequencing and bioinformatic analytical tools, opportunity to identify mechanosensitive TFs in cartilage is increased. By comparing the transcriptome differences in cartilage isolated from damaged distal medial condyle and intact posterior lateral condyle of femurs, Dunn et al. identified several key TFs (*FOSL1*, *AHR*, *E2F1*, and *FOXM1*) with links to a large proportion of differentially expressed genes in damaged cartilage. *FOSL1* was shown to be activated in loaded mandibular cartilage, suggesting that these four TFs may also participate in mechanotransduction processes in chondrocytes.^83,84^ More in-depth studies are required to identify additional TFs that participate in mechanotransduction.

To date, relevant studies to fully reveal the relationship between these TFs and the mechanosensors that we have listed above are lacking. We tentatively ascribe mechanotransduction activity to these TFs to be the expectation that future research will reveal additional comprehensive molecular pathways, which may help in better understanding the process of mechanoresponsiveness in chondrocytes. Notably, the abovementioned TFs are not exclusively activated in response to mechanical loading. When studying their mechanics-related function and interpreting experimental results, it is critical to consider the influence of other factors. For example, NF-κB and YAP are also regulated by inflammation and mitogenic growth factors, respectively.

## Role of ERα in regulating chondrocyte phenotype and responses to mechanical force

### ERα activation

**Fig. 4:**
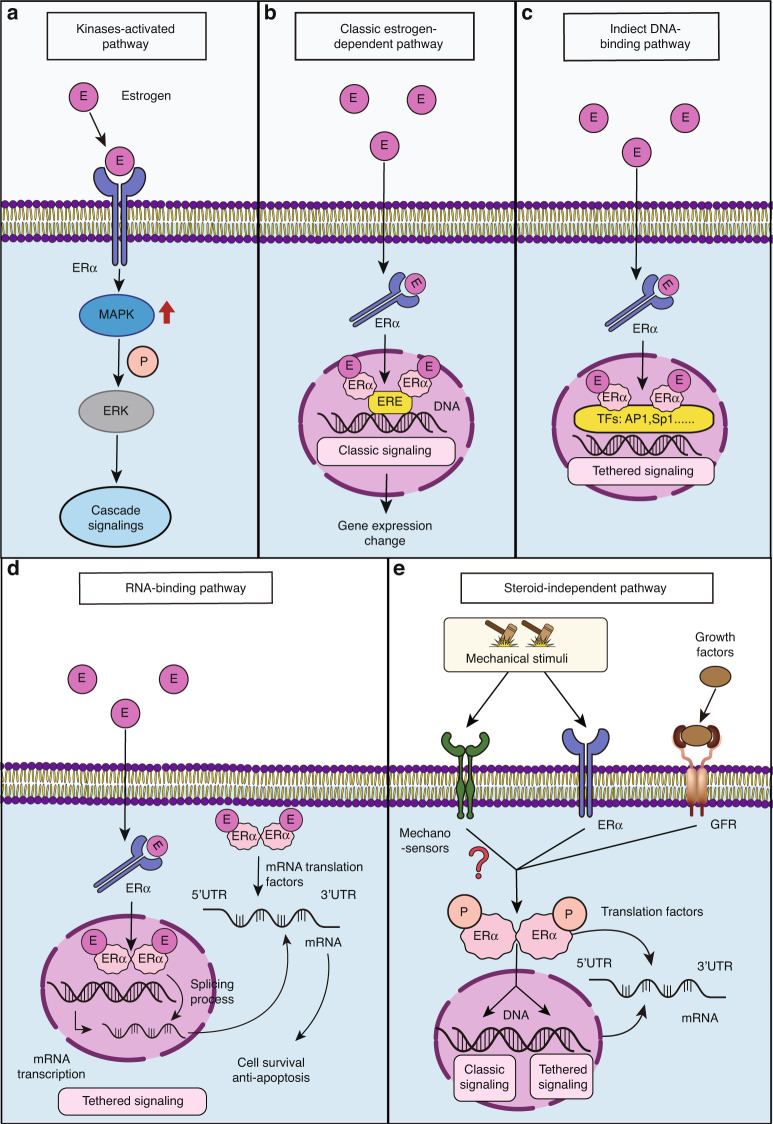
Known functions of ERα. **a–d** *Ligand-dependent regulatory pathways*. ERα binds with ligands such as E2, which can (**a**) directly activate MAPK signaling, **b** bind with DNA to function as a transcription factor (classic signaling factor), **c** bind with other transcription factors to influence gene expression (tethered signaling), and **d** modulate mRNA splicing or translational processes by binding to mRNA. **e** *Steroid-independent regulatory* *pathways.* ERα can be activated by mechanical load or several growth factors, E: estrogen, ERα: estrogen receptor α, MAPK: mitogen-activated protein kinase, ERK: extracellular signal-regulated kinase, P: phosphorylation, ERE: estrogen response element, TF: transcription factor, AP1: activating protein-1, Sp1: specificity protein 1, and GFR: growth factor receptor

The structure of ERα is composed of five domains, which include a DNA-binding domain, a ligand-binding domain, a DNA hinge, and two domains of activation functions (AF1 and AF2); AF1 is located at the N-terminus, and AF2 is adjacent to the ligand-binding domain.^85^ ERα is a well characterized receptor of estrogens, such as the sexual hormone 17β‐Estradiol (E2), but it can also be activated in a steroid-independent manner, including in a certain growth factor-dependent manner and ligand-independent manner (Fig. 4). Estrogen-related drugs for OA management and steroid-dependent functions of ERα in chondrocytes have were summarized by Xiao et al.^86–88^ In the present review, we discuss the potential of direct phosphorylation of ERα in a ligand-independent mechanism, specifically a mechanism triggered by mechanical load.^12^

### Expression of ERα in normal and OA cartilage

While female animals present with clearly higher levels of circulating estrogen-related ligands, recent studies have revealed that ERα is highly expressed in articular cartilage in animals of both sexes. Claassen et al. compared ERα protein levels in articular cartilage from cows, pigs, and humans after immunohistochemical staining and revealed that ERα was expressed in chondrocytes from different species.^89^ Oshima et al. also found that ERα was expressed in the articular cartilage and subchondral bone layers of adult rats of both sexes.^90^ Importantly, ERα was highly expressed in immature rats and expressed at lower levels in ovariectomized rats as well as aged rats, highlighting that ERα level changes were influenced by estrogen levels and aging.

Genetic analyses have revealed associations between alterations in *ESR1*, the gene encoding ERα, and osteoarthritis. For example, Ryder et al. conducted a systematic review of genetic association studies on OA. They found that ESR1 and AGC1, among other genes, showed a significant association with OA in 14 independent studies.^91^ Moreover, polymorphisms considered to be haplotype alleles of ESR1 [px (54%), PX (34%), and Px (12%)] were shown to be associated with an increased prevalence of radiographic knee OA, in particular in the presence of osteophytes.^92,93^ Another case‒control study investigated the association of ERα gene polymorphisms in patients with primary knee OA and healthy controls in the Korean population and found that the allele frequency for the exon 8G/A BtgI polymorphism (codon 594) was significantly different between patients with primary knee OA and individuals serving as controls (odds ratio = 1.38, 95% confidence interval = 1.01–1.88; *P* = 0.044).^94^ Moreover, the *ESR1* gene SNP rs2234693 was significantly associated with KOA in a dominant genetic model (TT + TC vs. CC) (odds ratio (OR) = 1.30; 95% confidence interval (CI) = 1.02–1.66; *P* = 0.03), and the T-allele frequency was also higher than that of allele C (OR = 1.38; 95% CI = 1.06–1.80; *P* = 0.02) in a 1953 subjects (1033 OA cases and 920 controls) from the Chinese Han population that were analyzed.^95^ Hence, genetic variations in the ERα gene have been associated with OA, especially in the presence of osteophytes.

Studies on the association between ERα levels and OA severity remain inconclusive. Ushiyama et al. extracted normal chondrocytes from the hip joint of femoral neck fracture patients and OA chondrocytes from the knee joint of TKA patients and compared the gene expression level of *ESR1* between these two groups via qRT‒PCR. No significant difference in *ESR1* gene expression was found between the cells from these joints.^96^ Notably, findings from this study may be of limited generalizability, as normal cartilage was harvested from a hip joint but not a knee joint, and the size of the healthy normal cartilage sample set was small. Several studies have reported RNA sequencing results used to compare the transcriptome differences in chondrocytes from normal cartilage (isolated from the knee joint of cadavers), mildly damaged cartilage (isolated from non-load-bearing cartilage areas in OA knees), and severely damaged cartilage (isolated from load-bearing cartilage areas in OA knees). *ESR1* showed the highest expression level in the normal cartilage and the lowest expression in OA cartilage from the load-bearing area (i.e., damaged cartilage).^84,97,98^ In particular, Wang et al. tested the protein expression level of ERα in preserved and damaged cartilage from human knee joints, as well as that from OA mouse models. The results showed that ERα was significantly decreased in damaged human cartilage and OA mouse knee joints, suggesting that ESR1 expression may be inhibited by mechanical overload and may be associated with OA pathology.^11^

The function of ERα in chondrocytes has also been studied through loss-of-function analysis. Sniekers et al. revealed that ERα^−/−^β^−/−^ mice presented with increased osteophytosis compared with that of their wild-type littermates, and deletion of either ERα or ERβ failed to induce cartilage damage or obvious osteophytosis.^99^ Wang et al. found loss of proteoglycan staining in ERα^−/−^ mice; however, these mice showed a degree of cartilage integrity maintenance that exceeded that in the controls.^11^ After applying cyclic mechanical loading to female osteoblast-specific-ERα-knockout (pOC-ERαKO) mice, Ziemian et al. found that the pOC-ERαKO mice developed more severe cartilage damage, larger osteophytes, and greater joint capsule fibrosis than the littermate controls, suggesting that loss of ERα in subchondral bone may be a potential mechanism of OA pathology.^100^ Engdahl et al. investigated the role of estrogen and ERα in a rheumatoid arthritis animal model established with cartilage-specific knockout (Col2a1-ERαKO) mice. They used antigen-induced arthritis to establish the model and analyzed knee joint synovitis and cartilage destruction. The results showed that ERα expression in cartilage was not required for estrogen-induced amelioration of joint destruction, but ERα expression in cartilage conferred protective effects against synovitis. These results suggest that ERα may play a role in inflammatory joint disease.^101^

To date, the mechanisms that result in the reduction of ERα levels in OA have not been fully determined. Our recent study demonstrated that mechanical overload caused decreases in ERα levels,^11^ implying that ERα may be highly responsive to stress. In fact, Guo et al. showed that IL-1β treatment induced a reduction in ERα levels, which was accompanied by elevated levels of miR-203 (ref. ^102^). Moreover, inhibiting miR-203 has been shown to restore ERα levels. Tian et al. performed bilateral ovariectomy and intra-articular monosodium iodoacetate injections (MIA) to establish a postmenopausal OA model in rats and found that the level of miR-203 was higher when the level of ERα was lower in OA knee joint cartilage. Similar to the in vitro study mentioned above, intra-articular injection of a miR-203 inhibitor attenuated cartilage degradation by elevating ERα levels in vivo.^103^ Overall, these results suggest that different OA-inducing stressors may reduce ERα levels in chondrocytes, and this effect is partially mediated by upregulated miR-203 activity.

### The ligand-independent mechanotransduction function of ERα

Our recent study revealed a ligand-independent mechanotransduction function for ERα in chondrocytes,^11^ and similar observations with other cell types has been previously reported. Rooney et al. reviewed mouse models used to study the role of ERα in bone and found that after ERα was depleted in differentiated osteoblasts, osteocytes, and osteoclasts in female mice, cancellous bone mass diminished.^104^ Zhang et al. found that mechanical loading stimulated cell proliferation and enhanced alkaline phosphatase activity in mesenchymal stem cells, noting that the effect was enhanced or inhibited by overexpressed and blocked ERα expression, respectively.^105^ Swift et al. revealed that mechanical loading significantly enhanced ERα expression in osteoblasts.^106^ However, according to the results from a study performed by Ehrlich et al., high mechanical strain decreased ERα expression.^107^ Overall, physiological loading appears to maintain a stable level of ERα, while damaging loads inhibit ERα expression.

Multiple independent studies have shown that the mechanotransduction role of ERα is at least partially estrogen independent. By stimulating the lower limbs of ovariectomized rats by mechanical overloading, the expression of ERα was significantly inhibited in bone, an effect that was independent of the estrogen concentration.^108^ Windahl et al. developed ERα AF1- or AF2-specific knockout mice and inserted a luciferase reporter into the estrogen response element (ERE) to determine whether the mechanotransduction of ERα in bone relied on estrogen.^12^ They found that the depletion of ERα AF1 blocked the effects of mechanical load on bone, but ERα AF2 knockout did not. Importantly, the luciferase reporter showed that ERα did not combine with the ERE to mediate the response to mechanical signals. In another study, Aguirre et al. first depleted ERα in osteocytes and osteoblasts, which impaired the cell response to stretching, and then transfected cells with an ERα mutant that could not bind estrogen. The results indicated that mutant ERα was sufficient to restore mechanoresponsiveness in these cells.^109^ In the same study, the researchers also demonstrated that the ligand-independent function necessarily involved ERα targeted to the plasma membrane, not to the nucleus. Zaman et al. also revealed specific strain-induced membrane localization of ERα in bone cells.^110^ Notably, the mechanoresponsiveness and ligand-independent function of ERα involved translocation from the cytoplasm to the nucleus, similar to the translocation of E2. Taken together, these data suggest that ERα exhibits a ligand-independent mechanotransduction function that requires its localization to the intact cell plasma membrane or cytoplasm, not the nucleus.

Recent findings have redefined the traditional concept of sex hormones as the main regulators of skeletal sexual dimorphism. For example, after aromatization-base transformation into estrogens, androgens activate not only the androgen receptor (AR) but also the ER. Sex hormone receptors exert an impact on the mechanical sensitivity of the growing skeleton.^111^ Therefore, even though sufficient evidence has shown that the mechanics-associated functions of ERα do not require classic ligands, very few studies have examined the effect when ligands are present. This effect is particularly important to women undergoing perimenopause. Although a simple experiment seems to be indicated, dissecting the functional changes of ERα based on the influence of decreased ERα levels after binding to estrogen is a challenge. Another important area for study is identification of the difference in DNA-binding sites in ERα in the presence and absence of estrogen. Potential ERα competition for classic estrogen responsive elements and other unknown responsive elements may ultimately affect the cell response to mechanical force.

### Downstream targets that are regulated by ERα in mechanotransduction pathways

To date, the targets of ERα activity contributing to mechanotransduction in chondrocytes have not been explored. Manolagas et al. reviewed the role played estrogen and androgen receptors in bone, suggesting that the most relevant downstream pathway of ERα involves WNT signaling, as ERα can sense mechanical loading and activate the WNT pathway to promote osteogenesis.^112^ Armstrong et al. also found that a single short period of dynamic mechanical strain increased the nuclear accumulation of activated beta-catenin. This effect was absent in primary osteoblast cultures from mice lacking ERα, suggesting that Wnt/beta-catenin signaling contributes to early responses to mechanical strain in cells, at least partially, through functional ERα.^113^ Under the stimulation of estrogen or loads, ERK-1, which belongs to the MAPK family, was activated after ERα was phosphorylated, demonstrating that ERα activates the MAPK/ERK-1 signaling pathway in response to mechanical loading.^114^ Another MAPK family member, JNK-1/2, is also involved in ERα signaling. Zhao et al. found that hydrostatic pressure was mediated through ERα to activate JNK-1/2 and in turn promoted the chondrogenic differentiation of MSCs.^115^ Last, Sunters et al. revealed that ERα facilitated the mechanical loading-activated signaling cascade in bone, which included insulin-like growth factor I receptor (IGF-IR), phosphatidylinositol 3-kinase-mediated phosphorylation of AKT, and then inhibited of GSK-3beta activity.^116^

**Fig. 5:**
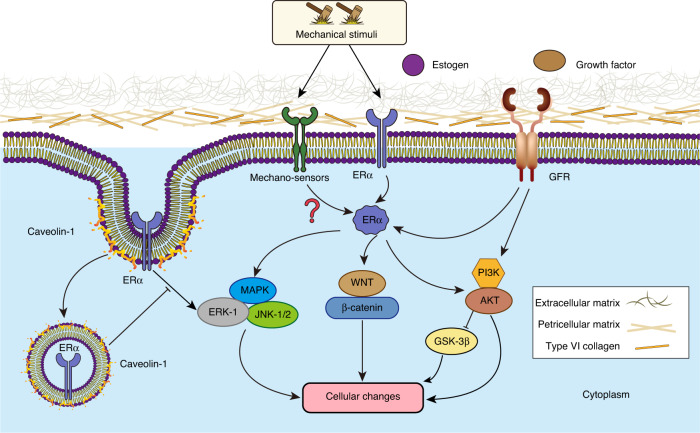
Potential targets that are regulated by ERα mediating chondrocyte responses to mechanical stimulation. Pointed arrows indicate stimulatory effects, and blocked lines indicate inhibitory effects. Some proposed pathways and interactions in chondrocytes remain to be determined. ERα: estrogen receptor α, MAPK: mitogen-activated protein kinase, ERK: extracellular signal-regulated kinase, JNK: c-Jun N-terminal kinase, GFR: growth factor receptor, PI3K: phosphatidylinositol-3-kinase, AKT: protein kinase B, and GSK-3β: glycogen synthase kinase 3 beta

Considering these findings in chondrocytes and other cell types, we propose a network that is regulated by ERα (Fig. 5), and this proposed pathway needs to be validated in future studies.

## Conclusion

Mechanical loading plays a fundamental role in the health and disease of articular cartilage. Physiological loading maintains the integrity of cartilage, while insufficient or excessive physical force can lead to its degradation. At present, a full molecular map by which physical forces are translated into biochemical responses remains to be established. Here, we summarize the current knowledge on the mechanotransduction pathways in articular chondrocytes. Inspired by previous studies, we propose a three-tiered cascade of mechanotransduction pathways to better characterize the role of these mechanoresponsive molecules and structures in chondrocytes. We believe that targeting mechanics-associated factor(s) is an important direction that will lead OA treatment because its converts the damaging effects of physical force and to be beneficial effects. Moreover, modulating these specific molecules to simulate mechanical signaling may not only allow physically disabled individuals to maintain healthy cartilage but also potentially regenerate/preserve cartilage in patients who have joint pain or restricted mobility; this possibility is particularly promising in the context of emerging concomitant cell therapies. In addition, by temporarily suppressing or activating key factors in the mechanotransduction pathway, it may also be possible to protect cartilage from damage due to short-term but high-intensity loading. For example, recent studies on TRPV4 and Piezo1/2 have indicated these possible outcomes, but the identification of the most efficacious targets is needed, and a greater number of more specific and efficacious small-molecule modulators are needed.

Notably, mechanical stimulation of articular cartilage induced by the movement of a joint involves very complex processes, including compression, shear stress, tension, hydrostatic pressure, and fluid movement.^117^ Moreover, the distribution and organization of cartilage components, including chondrocytes, various types of collagens, proteoglycans, noncollagenous proteins, and water, varies among the four distinct cartilage layers: The superficial, middle, deep, and calcified zones. All these objective reasons indicate that different mechanical effects on chondrocytes are mediated differentially by different layers.^51^ Due to the limitation in simulating the complexity of the native environment, most research has explored one type of mechanical stimulus without including the variation inherent in different cartilage zones. Although the simplified conditions have helped us understand mechanotransduction in chondrocytes, more physiologically relevant conditions are needed to investigate how chondrocytes sense different types of mechanical stimulus signals and convert them into biochemical changes. Moreover, as described above, most currently identified mechanically responsive transcription factors also participate in other cellular activities. Therefore, the degree of mechanotransduction pathway uniqueness in chondrocytes needs to be further investigated, which may include identifying unique transcription factors and kinases, discovering special molecular interactions that are observed only when a mechanical signal is applied, and identifying factors that can exclusively lead to an outcome on the basis of mechanical stimulation. This information will be critical to developing mechanics-regulated biomaterials and drugs in the future.^10^

An important topic that we need to specifically address in the future is sex-dependent differences in mechanotransduction pathways, which have often been overlooked. Using bovine explants, Hernandez showed that male and female cartilage tissues exhibit different mechanical properties, which are associated with different pericellular matrix compositions.^118^ Yu et al. compared the ERα level in articular chondrocytes of male and female rats at different ages. The results revealed that the ERα expression level and the number of ERα-positive chondrocytes remained stable in 4-weeks-old male rats until they were 12 months old, yet the aforementioned index significantly decreased with age in female rats.^119^ This study proved that the decrease in the level of ERα under the influence of aging was more pronounced in females. Whether a sex-dependent difference with the decrease ERα level results in a different responses to mechanical force between old male and female chondrocytes, however, remains to be reported. Melville et al. previously discovered that ERα in mature osteoblasts differentially regulated bone mass in males and females.^120^ It is therefore suggested that chondrocytes and animals from both sexes be included in future studies to determine the potential sex-dependent difference in mechanotransduction pathways, which will be critical to developing disease-modifying drugs. Overall, current evidence suggests that sexual dimorphism is not just a result of the discrepancy in sex steroid secretions between the sexes; in contrast, it depends on sex-dependent differences in the cellular response to mechanical forces.

In our previous study, we discovered that ERα is a negative regulator of p16^INK4a^, a marker of senescence, and that restoring the ERα level directly reduced the p16^INK4a^ level.^11^ Given that different stressors, such as mechanical overload, DNA damage, and inflammation, cause senescence, we speculate that ERα levels are influenced by these stressors and mediate the development of senescence in chondrocytes. In fact, our previous data showed that mechanical overload, one type of stressor that damages cartilage, led to decreased ERα levels.^11^ Therefore, ERα is thought to be a hub connecting senescence and mechanotransduction pathways. Future studies are needed to position ERα and its interactions in the context of other factors in these pathways.

Last, our current studies suggest that the role of ERα in influencing mechanotransduction in chondrocytes does not require the presence of estrogen. However, it is not clear whether the presence of estrogen affects the ligand-independent functions of ERα. If it does, how do chondrocytes interpret and integrate these two activation mechanisms? Estrogen therapy has been evaluated in human clinical trials to treat osteoarthritis. As discussed above, the binding of estrogen or selective estrogen receptor modulators resulted in the rapid degradation of ERα. If OA chondrocytes lose the capacity to sufficiently produce new ERα, they may undergo senescence conversion due to ligand-induced decreases in ERα levels. Therefore, it is important to delineate the ligand-dependent and ligand-independent functions of ERα in chondrocytes.

## References

[1] Loeser RF, Goldring SR, Scanzello CR, Goldring MB (2012). Osteoarthritis: a disease of the joint as an organ. Arthritis Rheum..

[2] Disease GBD, Injury I, Prevalence C (2018). Global, regional, and national incidence, prevalence, and years lived with disability for 354 diseases and injuries for 195 countries and territories, 1990–2017: a systematic analysis for the Global Burden of Disease Study 2017. Lancet.

[3] Martel-Pelletier J (2016). Osteoarthritis. Nat. Rev. Dis. Prim..

[4] Chang SH (2019). Excessive mechanical loading promotes osteoarthritis through the gremlin-1-NF-kappaB pathway. Nat. Commun..

[5] Lequesne MG, Dang N, Lane NE (1997). Sport practice and osteoarthritis of the limbs. Osteoarthr. Cartil..

[6] Griffin TM, Guilak F (2005). The role of mechanical loading in the onset and progression of osteoarthritis. Exerc Sport Sci. Rev..

[7] Kovar PA (1992). Supervised fitness walking in patients with osteoarthritis of the knee. A randomized, controlled trial. Ann. Intern. Med..

[8] Allen J (2017). Effects of treadmill exercise on advanced osteoarthritis pain in rats. Arthritis Rheumatol..

[9] Bannuru RR (2019). OARSI guidelines for the non-surgical management of knee, hip, and polyarticular osteoarthritis. Osteoarthr. Cartil..

[10] Hodgkinson T, Kelly DC, Curtin CM, O’Brien FJ (2022). Mechanosignalling in cartilage: an emerging target for the treatment of osteoarthritis. Nat. Rev. Rheumatol..

[11] Wang N (2022). Novel role of estrogen receptor-alpha on regulating chondrocyte phenotype and response to mechanical loading. Osteoarthr. Cartil..

[12] Windahl SH (2013). Estrogen receptor-alpha is required for the osteogenic response to mechanical loading in a ligand-independent manner involving its activation function 1 but not 2. J. Bone Min. Res..

[13] Gilbert SJ, Bonnet CS, Blain EJ (2021). Mechanical cues: bidirectional reciprocity in the extracellular matrix drives mechano-signalling in articular cartilage. Int. J. Mol. Sci..

[14] Dieterle MP, Husari A, Rolauffs B, Steinberg T, Tomakidi P (2021). Integrins, cadherins and channels in cartilage mechanotransduction: perspectives for future regeneration strategies. Expert Rev. Mol. Med..

[15] Wang L (2020). Mechanical sensing protein PIEZO1 regulates bone homeostasis via osteoblast-osteoclast crosstalk. Nat. Commun..

[16] Pathak MM (2014). Stretch-activated ion channel Piezo1 directs lineage choice in human neural stem cells. Proc. Natl. Acad. Sci. USA.

[17] Panciera T, Azzolin L, Cordenonsi M, Piccolo S (2017). Mechanobiology of YAP and TAZ in physiology and disease. Nat. Rev. Mol. Cell Biol..

[18] Fermor B (2002). Induction of cyclooxygenase-2 by mechanical stress through a nitric oxide-regulated pathway. Osteoarthr. Cartil..

[19] O’Conor CJ, Leddy HA, Benefield HC, Liedtke WB, Guilak F (2014). TRPV4-mediated mechanotransduction regulates the metabolic response of chondrocytes to dynamic loading. Proc. Natl. Acad. Sci. USA.

[20] Kawakita K (2012). Akt phosphorylation in human chondrocytes is regulated by p53R2 in response to mechanical stress. Osteoarthr. Cartil..

[21] Gudipaty SA (2017). Mechanical stretch triggers rapid epithelial cell division through Piezo1. Nature.

[22] Zeng WZ (2018). PIEZOs mediate neuronal sensing of blood pressure and the baroreceptor reflex. Science.

[23] Delco ML, Bonassar LJ (2021). Targeting calcium-related mechanotransduction in early OA. Nat. Rev. Rheumatol..

[24] Phan MN (2009). Functional characterization of TRPV4 as an osmotically sensitive ion channel in porcine articular chondrocytes. Arthritis Rheum..

[25] Clark AL, Votta BJ, Kumar S, Liedtke W, Guilak F (2010). Chondroprotective role of the osmotically sensitive ion channel transient receptor potential vanilloid 4: age- and sex-dependent progression of osteoarthritis in Trpv4-deficient mice. Arthritis Rheum..

[26] Fu S (2021). Activation of TRPV4 by mechanical, osmotic or pharmaceutical stimulation is anti-inflammatory blocking IL-1beta mediated articular cartilage matrix destruction. Osteoarthr. Cartil..

[27] Agarwal P (2021). A dysfunctional TRPV4-GSK3beta pathway prevents osteoarthritic chondrocytes from sensing changes in extracellular matrix viscoelasticity. Nat. Biomed. Eng..

[28] Lee W (2014). Synergy between Piezo1 and Piezo2 channels confers high-strain mechanosensitivity to articular cartilage. Proc. Natl. Acad. Sci. USA.

[29] Servin-Vences MR, Moroni M, Lewin GR, Poole K (2017). Direct measurement of TRPV4 and PIEZO1 activity reveals multiple mechanotransduction pathways in chondrocytes. Elife.

[30] Lee W (2021). Inflammatory signaling sensitizes Piezo1 mechanotransduction in articular chondrocytes as a pathogenic feed-forward mechanism in osteoarthritis. Proc. Natl. Acad. Sci. USA.

[31] Du G (2020). Roles of TRPV4 and piezo channels in stretch-evoked Ca(2+) response in chondrocytes. Exp. Biol. Med. (Maywood).

[32] Li, X. F., Zhang, Z., Li, X. D., Wang, T. B. & Zhang, H. N. [Mechanism of the Piezo1 protein-induced apoptosis of the chondrocytes through the MAPK/ERK1/2 signal pathway]. *Zhonghua yi xue za zhi* **96**, 2472–2477 (2016). 10.3760/cma.j.issn.0376-2491.2016.31.007 27562045

[33] Thompson CL, Chapple JP, Knight MM (2014). Primary cilia disassembly down-regulates mechanosensitive hedgehog signalling: a feedback mechanism controlling ADAMTS-5 expression in chondrocytes. Osteoarthr. Cartil..

[34] Ruhlen R, Marberry K (2014). The chondrocyte primary cilium. Osteoarthr. Cartil..

[35] Wann AK, Knight MM (2012). Primary cilia elongation in response to interleukin-1 mediates the inflammatory response. Cell. Mol. Life Sci..

[36] He Z (2016). Strain-induced mechanotransduction through primary cilia, extracellular ATP, purinergic calcium signaling, and ERK1/2 transactivates CITED2 and downregulates MMP-1 and MMP-13 gene expression in chondrocytes. Osteoarthr. Cartil..

[37] Juhasz T (2014). Mechanical loading stimulates chondrogenesis via the PKA/CREB-Sox9 and PP2A pathways in chicken micromass cultures. Cell Signal.

[38] Kwon RY, Temiyasathit S, Tummala P, Quah CC, Jacobs CR (2010). Primary cilium-dependent mechanosensing is mediated by adenylyl cyclase 6 and cyclic AMP in bone cells. FASEB J..

[39] Wang Q (2019). Dysregulated integrin alphaVbeta3 and CD47 signaling promotes joint inflammation, cartilage breakdown, and progression of osteoarthritis. JCI Insight.

[40] Loeser RF (2014). Integrins and chondrocyte-matrix interactions in articular cartilage. Matrix Biol..

[41] Zhen G (2021). Mechanical stress determines the configuration of TGFbeta activation in articular cartilage. Nat. Commun..

[42] Zhou Y (2007). Evidence for JNK-dependent up-regulation of proteoglycan synthesis and for activation of JNK1 following cyclical mechanical stimulation in a human chondrocyte culture model. Osteoarthr. Cartil..

[43] Ma D (2016). Hydrostatic compress force enhances the viability and decreases the apoptosis of condylar chondrocytes through integrin-FAK-ERK/PI3K pathway. Int. J. Mol. Sci..

[44] Millward-Sadler SJ (1999). Integrin-regulated secretion of interleukin 4: a novel pathway of mechanotransduction in human articular chondrocytes. J. Cell Biol..

[45] Shingu M, Miyauchi S, Nagai Y, Yasutake C, Horie K (1995). The role of IL-4 and IL-6 in IL-1-dependent cartilage matrix degradation. Br. J. Rheumatol..

[46] Nemoto O (1997). Suppression of matrix metalloproteinase-3 synthesis by interleukin-4 in human articular chondrocytes. J. Rheumatol..

[47] Millward-Sadler SJ, Wright MO, Davies LW, Nuki G, Salter DM (2000). Mechanotransduction via integrins and interleukin-4 results in altered aggrecan and matrix metalloproteinase 3 gene expression in normal, but not osteoarthritic, human articular chondrocytes. Arthritis Rheum..

[48] Poole CA, Flint MH, Beaumont BW (1987). Chondrons in cartilage: ultrastructural analysis of the pericellular microenvironment in adult human articular cartilages. J. Orthop. Res..

[49] Guilak F, Nims RJ, Dicks A, Wu CL, Meulenbelt I (2018). Osteoarthritis as a disease of the cartilage pericellular matrix. Matrix Biol..

[50] Zelenski NA (2015). Type VI collagen regulates pericellular matrix properties, chondrocyte swelling, and mechanotransduction in mouse articular cartilage. Arthritis Rheumatol..

[51] Browne JE, Branch TP (2000). Surgical alternatives for treatment of articular cartilage lesions. J. Am. Acad. Orthop. Surg..

[52] Guilak F (1995). Compression-induced changes in the shape and volume of the chondrocyte nucleus. J. Biomech..

[53] Ghosh S (2019). Deformation microscopy for dynamic intracellular and intranuclear mapping of mechanics with high spatiotemporal resolution. Cell Rep..

[54] Wolff KJ (2013). Mechanical stress and ATP synthesis are coupled by mitochondrial oxidants in articular cartilage. J. Orthop. Res..

[55] Szafranski JD (2004). Chondrocyte mechanotransduction: effects of compression on deformation of intracellular organelles and relevance to cellular biosynthesis. Osteoarthr. Cartil..

[56] Irianto J (2013). Osmotic challenge drives rapid and reversible chromatin condensation in chondrocytes. Biophys. J..

[57] Reynolds N (2021). Image-derived modeling of nucleus strain amplification associated with chromatin heterogeneity. Biophys. J..

[58] Chao PH, West AC, Hung CT (2006). Chondrocyte intracellular calcium, cytoskeletal organization, and gene expression responses to dynamic osmotic loading. Am. J. Physiol. Cell Physiol..

[59] Erickson GR, Northrup DL, Guilak F (2003). Hypo-osmotic stress induces calcium-dependent actin reorganization in articular chondrocytes. Osteoarthr. Cartil..

[60] Lauer JC, Selig M, Hart ML, Kurz B, Rolauffs B (2021). Articular chondrocyte phenotype regulation through the cytoskeleton and the signaling processes that originate from or converge on the cytoskeleton: towards a novel understanding of the intersection between actin dynamics and chondrogenic function. Int. J. Mol. Sci..

[61] Jiang W (2021). Mechanisms linking mitochondrial mechanotransduction and chondrocyte biology in the pathogenesis of osteoarthritis. Ageing Res. Rev..

[62] He Y, Makarczyk MJ, Lin H (2020). Role of mitochondria in mediating chondrocyte response to mechanical stimuli. Life Sci..

[63] Sauter E, Buckwalter JA, McKinley TO, Martin JA (2012). Cytoskeletal dissolution blocks oxidant release and cell death in injured cartilage. J. Orthop. Res..

[64] Bartell LR (2020). Mitoprotective therapy prevents rapid, strain-dependent mitochondrial dysfunction after articular cartilage injury. J. Orthop. Res..

[65] Koike M (2015). Mechanical overloading causes mitochondrial superoxide and SOD2 imbalance in chondrocytes resulting in cartilage degeneration. Sci. Rep..

[66] Buckwalter JA, Anderson DD, Brown TD, Tochigi Y, Martin JA (2013). The roles of mechanical stresses in the pathogenesis of osteoarthritis: implications for treatment of joint injuries. Cartilage.

[67] Oeckinghaus A, Ghosh S (2009). The NF-kappaB family of transcription factors and its regulation. Cold Spring Harb. Perspect. Biol..

[68] Liu T, Zhang L, Joo D, Sun SC (2017). NF-kappaB signaling in inflammation. Signal Transduct. Target Ther..

[69] Nam J, Aguda BD, Rath B, Agarwal S (2009). Biomechanical thresholds regulate inflammation through the NF-kappaB pathway: experiments and modeling. PLoS One.

[70] Chen NX, Geist DJ, Genetos DC, Pavalko FM, Duncan RL (2003). Fluid shear-induced NFkappaB translocation in osteoblasts is mediated by intracellular calcium release. Bone.

[71] Khachigian LM, Resnick N, Gimbrone MA, Collins T (1995). Nuclear factor-kappa B interacts functionally with the platelet-derived growth factor B-chain shear-stress response element in vascular endothelial cells exposed to fluid shear stress. J. Clin. Investig..

[72] Tong L, Tergaonkar V (2014). Rho protein GTPases and their interactions with NFkappaB: crossroads of inflammation and matrix biology. Biosci. Rep..

[73] Shrum CK, Defrancisco D, Meffert MK (2009). Stimulated nuclear translocation of NF-kappaB and shuttling differentially depend on dynein and the dynactin complex. Proc. Natl. Acad. Sci. USA.

[74] Mikenberg I, Widera D, Kaus A, Kaltschmidt B, Kaltschmidt C (2007). Transcription factor NF-kappaB is transported to the nucleus via cytoplasmic dynein/dynactin motor complex in hippocampal neurons. PLoS One.

[75] Rai A, Kapoor S, Singh S, Chatterji BP, Panda D (2015). Transcription factor NF-kappaB associates with microtubules and stimulates apoptosis in response to suppression of microtubule dynamics in MCF-7 cells. Biochem. Pharm..

[76] Nemeth ZH (2004). Disruption of the actin cytoskeleton results in nuclear factor-kappaB activation and inflammatory mediator production in cultured human intestinal epithelial cells. J. Cell Physiol..

[77] Yang Y (2019). Mechanical stress protects against osteoarthritis via regulation of the AMPK/NF-kappaB signaling pathway. J. Cell Physiol..

[78] Yang Y (2020). Moderate mechanical stimulation protects rats against osteoarthritis through the regulation of TRAIL via the NF-kappaB/NLRP3 pathway. Oxid. Med. Cell Longev..

[79] Xie M (2021). Dynamic loading enhances chondrogenesis of human chondrocytes within a biodegradable resilient hydrogel. Biomater. Sci..

[80] Sun Y (2021). G protein coupled estrogen receptor attenuates mechanical stress-mediated apoptosis of chondrocyte in osteoarthritis via suppression of Piezo1. Mol. Med..

[81] Zhang X (2020). Targeting downstream subcellular YAP activity as a function of matrix stiffness with Verteporfin-encapsulated chitosan microsphere attenuates osteoarthritis. Biomaterials.

[82] Xuan F (2019). Wnt/beta-catenin signaling contributes to articular cartilage homeostasis through lubricin induction in the superficial zone. Arthritis Res. Ther..

[83] Papachristou D, Pirttiniemi P, Kantomaa T, Agnantis N, Basdra EK (2006). Fos- and Jun-related transcription factors are involved in the signal transduction pathway of mechanical loading in condylar chondrocytes. Eur. J. Orthod..

[84] Dunn SL (2016). Gene expression changes in damaged osteoarthritic cartilage identify a signature of non-chondrogenic and mechanical responses. Osteoarthr. Cartil..

[85] Borjesson AE (2011). Roles of transactivating functions 1 and 2 of estrogen receptor-alpha in bone. Proc. Natl. Acad. Sci. USA.

[86] Xiao YP (2016). Are estrogen-related drugs new alternatives for the management of osteoarthritis. Arthritis Res. Ther..

[87] Kato S (1995). Activation of the estrogen receptor through phosphorylation by mitogen-activated protein kinase. Science.

[88] Bunone G, Briand PA, Miksicek RJ, Picard D (1996). Activation of the unliganded estrogen receptor by EGF involves the MAP kinase pathway and direct phosphorylation. EMBO J..

[89] Claassen H (2001). Immunohistochemical detection of estrogen receptor alpha in articular chondrocytes from cows, pigs and humans: in situ and in vitro results. Ann. Anat..

[90] Oshima Y (2007). Localization of estrogen receptors alpha and beta in the articular surface of the rat femur. Acta Histochem. Cytochem..

[91] Ryder JJ (2008). Genetic associations in peripheral joint osteoarthritis and spinal degenerative disease: a systematic review. Ann. Rheum. Dis..

[92] Valdes AM (2004). Association study of candidate genes for the prevalence and progression of knee osteoarthritis. Arthritis Rheum..

[93] Bergink AP (2003). Estrogen receptor alpha gene haplotype is associated with radiographic osteoarthritis of the knee in elderly men and women. Arthritis Rheum..

[94] Jin SY (2004). Estrogen receptor-alpha gene haplotype is associated with primary knee osteoarthritis in Korean population. Arthritis Res. Ther..

[95] Dai X (2020). Genetic estrogen receptor alpha gene PvuII polymorphism in susceptibility to knee osteoarthritis in a Chinese Han population: A southern Jiangsu study. Knee.

[96] Ushiyama T, Ueyama H, Inoue K, Ohkubo I, Hukuda S (1999). Expression of genes for estrogen receptors alpha and beta in human articular chondrocytes. Osteoarthr. Cartil..

[97] Wang B (2022). Exploring the mystery of osteoarthritis using bioinformatics analysis of cartilage tissue. Comb. Chem. High. Throughput Screen.

[98] Ramos YF (2014). Genes involved in the osteoarthritis process identified through genome wide expression analysis in articular cartilage; the RAAK study. PLoS One.

[99] Sniekers YH (2009). Development of osteoarthritic features in estrogen receptor knockout mice. Osteoarthr. Cartil..

[100] Ziemian SN (2021). Low bone mass resulting from impaired estrogen signaling in bone increases severity of load-induced osteoarthritis in female mice. Bone.

[101] Engdahl C (2014). The role of total and cartilage-specific estrogen receptor alpha expression for the ameliorating effect of estrogen treatment on arthritis. Arthritis Res. Ther..

[102] Guo Y, Tian L, Du X, Deng Z (2020). MiR-203 regulates estrogen receptor alpha and cartilage degradation in IL-1beta-stimulated chondrocytes. J. Bone Min. Metab..

[103] Tian L, Su Z, Ma X, Wang F, Guo Y (2019). Inhibition of miR-203 ameliorates osteoarthritis cartilage degradation in the postmenopausal rat model: involvement of estrogen receptor alpha. Hum. Gene Ther. Clin. Dev..

[104] Rooney AM, van der Meulen MCH (2017). Mouse models to evaluate the role of estrogen receptor alpha in skeletal maintenance and adaptation. Ann. N. Y. Acad. Sci..

[105] Zhang M (2012). Estrogen and its receptor enhance mechanobiological effects in compressed bone mesenchymal stem cells. Cells Tissues Organs.

[106] Swift SN, Swift JM, Bloomfield SA (2014). Mechanical loading increases detection of estrogen receptor-alpha in osteocytes and osteoblasts despite chronic energy restriction. J. Appl. Physiol. (1985).

[107] Ehrlich PJ (2002). The effect of in vivo mechanical loading on estrogen receptor alpha expression in rat ulnar osteocytes. J. Bone Min. Res..

[108] Nepal AK (2021). Mechanical stress regulates bone regulatory gene expression independent of estrogen and vitamin D deficiency in rats. J. Orthop. Res..

[109] Aguirre JI (2007). A novel ligand-independent function of the estrogen receptor is essential for osteocyte and osteoblast mechanotransduction. J. Biol. Chem..

[110] Zaman G (2006). Osteocytes use estrogen receptor alpha to respond to strain but their ERalpha content is regulated by estrogen. J. Bone Min. Res..

[111] Callewaert F, Sinnesael M, Gielen E, Boonen S, Vanderschueren D (2010). Skeletal sexual dimorphism: relative contribution of sex steroids, GH-IGF1, and mechanical loading. J. Endocrinol..

[112] Manolagas SC, O’Brien CA, Almeida M (2013). The role of estrogen and androgen receptors in bone health and disease. Nat. Rev. Endocrinol..

[113] Armstrong VJ (2007). Wnt/beta-catenin signaling is a component of osteoblastic bone cell early responses to load-bearing and requires estrogen receptor alpha. J. Biol. Chem..

[114] Jessop HL (2001). Mechanical strain and estrogen activate estrogen receptor alpha in bone cells. J. Bone Min. Res..

[115] Zhao Y (2016). The distinct effects of estrogen and hydrostatic pressure on mesenchymal stem cells differentiation: involvement of estrogen receptor signaling. Ann. Biomed. Eng..

[116] Sunters A (2010). Mechano-transduction in osteoblastic cells involves strain-regulated estrogen receptor alpha-mediated control of insulin-like growth factor (IGF) I receptor sensitivity to Ambient IGF, leading to phosphatidylinositol 3-kinase/AKT-dependent Wnt/LRP5 receptor-independent activation of beta-catenin signaling. J. Biol. Chem..

[117] Uzieliene I, Bironaite D, Bernotas P, Sobolev A, Bernotiene E (2021). Mechanotransducive biomimetic systems for chondrogenic differentiation in vitro. Int. J. Mol. Sci..

[118] Hernandez PA (2022). Sexual dimorphism in the extracellular and pericellular matrix of articular cartilage. Cartilage.

[119] Yu SB (2009). The effects of age and sex on the expression of oestrogen and its receptors in rat mandibular condylar cartilages. Arch. Oral. Biol..

[120] Melville KM (2015). Effects of deletion of ERalpha in osteoblast-lineage cells on bone mass and adaptation to mechanical loading differ in female and male mice. J. Bone Min. Res..

[121] Xu B (2019). Excessive mechanical stress induces chondrocyte apoptosis through TRPV4 in an anterior cruciate ligament-transected rat osteoarthritis model. Life Sci..

[122] O’Conor CJ (2016). Cartilage-specific knockout of the mechanosensory ion channel TRPV4 decreases age-related osteoarthritis. Sci. Rep..

[123] Sun Y (2020). Mechanism of abnormal chondrocyte proliferation induced by piezo1-siRNA exposed to mechanical stretch. Biomed. Res. Int..

[124] Chang CF, Ramaswamy G, Serra R (2012). Depletion of primary cilia in articular chondrocytes results in reduced Gli3 repressor to activator ratio, increased Hedgehog signaling, and symptoms of early osteoarthritis. Osteoarthr. Cartil..

[125] Irianto J, Ramaswamy G, Serra R, Knight MM (2014). Depletion of chondrocyte primary cilia reduces the compressive modulus of articular cartilage. J. Biomech..

[126] Yuan X, Yang S (2015). Deletion of IFT80 impairs epiphyseal and articular cartilage formation due to disruption of chondrocyte differentiation. PLoS One.

[127] Almonte-Becerril M (2018). Genetic abrogation of the fibronectin-alpha5beta1 integrin interaction in articular cartilage aggravates osteoarthritis in mice. PLoS One.

[128] Chery DR (2021). Decorin regulates cartilage pericellular matrix micromechanobiology. Matrix Biol..

[129] Fioravanti A, Benetti D, Coppola G, Collodel G (2005). Effect of continuous high hydrostatic pressure on the morphology and cytoskeleton of normal and osteoarthritic human chondrocytes cultivated in alginate gels. Clin. Exp. Rheumatol..

[130] Chen C (2016). Effects of vimentin disruption on the mechanoresponses of articular chondrocyte. Biochem. Biophys. Res. Commun..

[131] Blain EJ, Mason DJ, Duance VC (2002). The effect of thymosin beta4 on articular cartilage chondrocyte matrix metalloproteinase expression. Biochem. Soc. Trans..

[132] Huser CA, Davies ME (2007). Calcium signaling leads to mitochondrial depolarization in impact-induced chondrocyte death in equine articular cartilage explants. Arthritis Rheum..

[133] Agarwal S (2004). Role of NF-kappaB transcription factors in antiinflammatory and proinflammatory actions of mechanical signals. Arthritis Rheum..

[134] Li W (2021). Role of HIF-2alpha/NF-kappaB pathway in mechanical stress-induced temporomandibular joint osteoarthritis. Oral. Dis..

[135] Jing X (2020). Mechanical loading induces HIF-1alpha expression in chondrocytes via YAP. Biotechnol. Lett..

[136] Deng Y (2018). Reciprocal inhibition of YAP/TAZ and NF-kappaB regulates osteoarthritic cartilage degradation. Nat. Commun..

[137] Tetsunaga T (2011). Regulation of mechanical stress-induced MMP-13 and ADAMTS-5 expression by RUNX-2 transcriptional factor in SW1353 chondrocyte-like cells. Osteoarthr. Cartil..

[138] Papachristou DJ, Pirttiniemi P, Kantomaa T, Papavassiliou AG, Basdra EK (2005). JNK/ERK-AP-1/Runx2 induction “paves the way” to cartilage load-ignited chondroblastic differentiation. Histochem. Cell Biol..

[139] Xiao L (2018). TGF-beta/SMAD signaling inhibits intermittent cyclic mechanical tension-induced degeneration of endplate chondrocytes by regulating the miR-455-5p/RUNX2 axis. J. Cell Biochem..

[140] De Croos JN, Dhaliwal SS, Grynpas MD, Pilliar RM, Kandel RA (2006). Cyclic compressive mechanical stimulation induces sequential catabolic and anabolic gene changes in chondrocytes resulting in increased extracellular matrix accumulation. Matrix Biol..

[141] Healy ZR, Zhu F, Stull JD, Konstantopoulos K (2008). Elucidation of the signaling network of COX-2 induction in sheared chondrocytes: COX-2 is induced via a Rac/MEKK1/MKK7/JNK2/c-Jun-C/EBPbeta-dependent pathway. Am. J. Physiol. Cell Physiol..

[142] De Croos JN, Pilliar RM, Kandel RA (2008). AP-1 DNA binding activity regulates the cartilage tissue remodeling process following cyclic compression in vitro. Biorheology.

[143] Furumatsu T (2013). Tensile strain increases expression of CCN2 and COL2A1 by activating TGF-beta-Smad2/3 pathway in chondrocytic cells. J. Biomech..

[144] Shen PC (2021). Shockwave treatment enhanced extracellular matrix production in articular chondrocytes through activation of the ROS/MAPK/Nrf2 signaling pathway. Cartilage.

[145] Thomas RS, Clarke AR, Duance VC, Blain EJ (2011). Effects of Wnt3A and mechanical load on cartilage chondrocyte homeostasis. Arthritis Res. Ther..

